# NSUN2 promotes osteosarcoma progression by enhancing the stability of FABP5 mRNA via m^5^C methylation

**DOI:** 10.1038/s41419-023-05646-x

**Published:** 2023-02-15

**Authors:** Min Yang, Renxiong Wei, Sheng Zhang, Sang Hu, Xiaoxiao Liang, Zhiqiang Yang, Chong Zhang, Yufeng Zhang, Lin Cai, Yuanlong Xie

**Affiliations:** 1 Department of Spine Surgery and Musculoskeletal Tumor, Zhongnan Hospital of Wuhan University, Wuhan, 430071 People’s Republic of China; 2 Department of Bone and Soft Tissue, Affiliated Tumor Hospital of Zhengzhou University, Zhengzhou, 450003 People’s Republic of China

**Keywords:** Bone cancer, Mechanisms of disease, Oncogenes, Tumour biomarkers

## Abstract

5-methylcytosine (m^5^C) modification, which is mainly induced by the RNA methyltransferase NSUN2 (NOP2/Sun domain family, member 2), is an important chemical posttranscriptional modification in mRNA and has been proven to play important roles in the progression of many cancers. However, the functions and underlying molecular mechanisms of NSUN2-mediated m^5^C in osteosarcoma (OS) remain unclear. In this study, we found NSUN2 was highly expressed in OS tissues and cells. We also discovered that higher expression of NSUN2 predicted poorer prognosis of OS patients. Our study showed that NSUN2 could promote the progression of OS cells. Moreover, we employed RNA sequencing, RNA immunoprecipitation (RIP), and methylated RIP to screen and validate the candidate targets of NSUN2 and identified FABP5 as the target. We observed that NSUN2 stabilized FABP5 mRNA by inducing m^5^C modification and further promoted fatty acid metabolism in OS cells. Moreover, both knocking down the expression of FABP5 and adding fatty acid oxidation inhibitor could counterbalance the promoting effect of NSUN2 on the progression of OS. Our study confirms that NSUN2 can up-regulate the expression of FABP5 by improving the stability of FABP5 mRNA via m^5^C, so as to promote fatty acid metabolism in OS cells, and finally plays the role in promoting the progression of OS. Our findings suggest that NSUN2 is a promising prognostic marker for OS patients and may serve as a potential therapeutic target for OS treatment.

**Figure:**
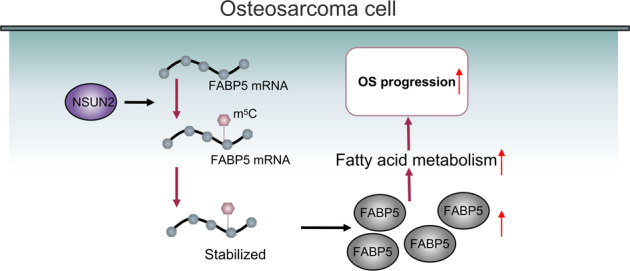
A schematic illustration was proposed to summarize our findings.

A schematic illustration was proposed to summarize our findings.

## Introduction

Osteosarcoma (OS) is the most common primary malignant bone cancer in children and adolescents, and has a high rate of death and disability. The standard treatment is neoadjuvant chemotherapy combined with surgical resection, but the overall survival rate of OS patients has remained at 60–70% [1, 2] for the last 30 years. Moreover, the side effects of chemotherapy cause great physical and mental harm to children and adolescents. Several clinical trials on immunotherapies for OS have failed to show encouraging therapeutic effects [3, 4]. Therefore, studying the pathological mechanism of OS may suggest a new way to treat OS.

RNA methylation modifications, such as N6-methyladenosine (m^6^A), have been reported to play crucial roles in regulating cellular mRNA metabolism and function in diverse disease processes [5–7]. Recently, the 5-methylcytosine (m^5^C) modification, which has been extensively studied in DNA, tRNA, and rRNA, has been shown to be related to mRNA metabolic regulation [8, 9]. Researchers have mapped large transcriptome-wide m^5^C profiles in multiple human and mouse tissues on the basis of advances in high-throughput approaches [10, 11], including RNA-bisulfite sequencing (RNA-BisSeq), m^5^C-RNA immunoprecipitation with next-generation sequencing (m^5^C-seq or MeRIP-seq) and methylation-individual-nucleotide-resolution crosslinking and immunoprecipitation (miCLIP). The deposition of m^5^C on mRNA has been reported to be catalyzed by NOP2/Sun RNA methyltransferase family members 2 and 6 (NSUN2 and NSUN6, respectively) and DNMT2 [12]. In contrast, TET2 and TET3 act as erasers that remove the methyl group from m^5^C-methylated bases [12]. Recently, Aly/REF export factor (ALYREF) and YBX1 have been identified as mRNA m^5^C-binding proteins (readers) [12]. According to published data, m^5^C modification exerts significant effects on mRNA metabolic processes; for example, it alters RNA stability, translation efficiency, and subcellular localization [13]. Thus, m^5^C on RNA is a widespread modification that may induce important cellular processes.

Several studies have revealed the crucial roles of m^5^C modification in human cancers and diseases. For instance, factors that regulate m^5^C RNA modification can predict the clinical prognostic risks of diverse human cancers. NSUN2, the main enzyme catalyzing m^5^C formation, has been demonstrated to promote tumorigenesis in bladder cancer [14], hepatocellular carcinoma [15], breast cancer [16], and gastric cancer [17] by regulating m^5^C modification at the transcriptome level. Tet2 deficiency causes the transcriptome-wide appearance of methylated cytosines, which may promote pathogen infection-induced myelopoiesis [18]. Therefore, m^5^C on mRNA may affect tumorigenesis and human cancer prognosis; however, the precise role of m^5^C modification in OS remains largely unstudied.

In this study, we found that NSUN2 was significantly upregulated in OS cells and tissues. In addition, higher NSUN2 expression predicted poorer prognosis in OS patients. Moreover, NSUN2 facilitated OS progression by enhancing FABP5 mRNA stability and then promoting fatty acid metabolism in OS cells, indicating that NSUN2 may be a potential therapeutic target in OS.

## Results

### NSUN2 is highly expressed in OS cells and tissues

**Fig. 1:**
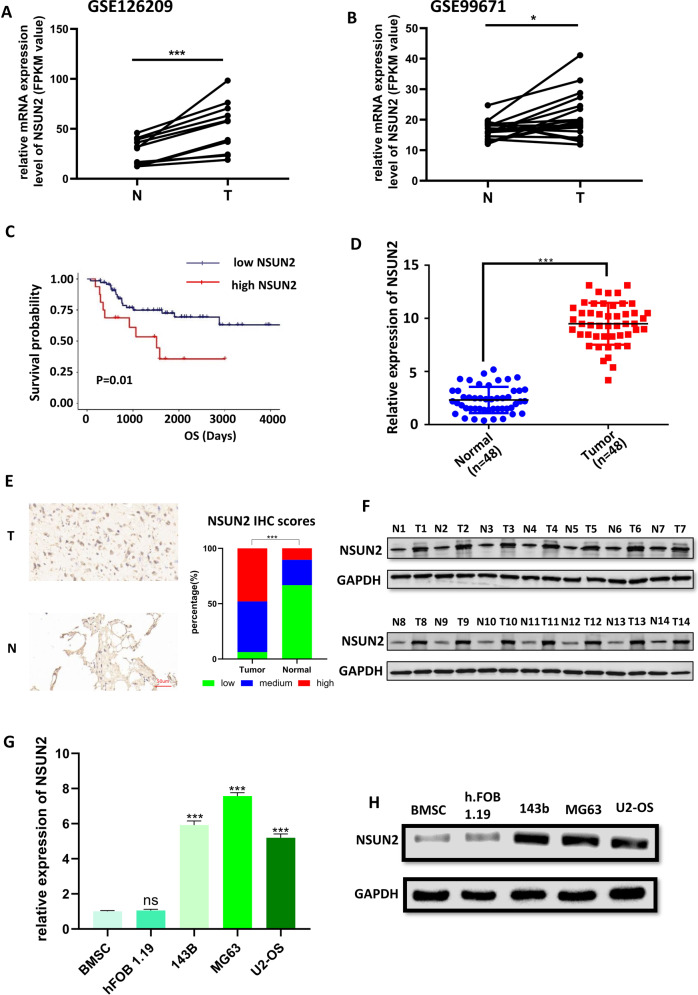
NSUN2 is highly expressed in OS cells and tissues. **A**, **B** The expression of NSUN2 in OS tissues and normal tissues from GSE126209 (**A**) and GSE99671 (**B**). **C** The survival analysis is made by the data from UCSC-XENA and the software R 3.6.2. The patients were divided into high-NSUN2 and low-NSUN2 by the software R 3.6.2. The results of RT-qPCR (**D**), IHC (**E**), and western blot (**F**) confirmed the expression of NSUN2 was higher in OS tissues. The results of RT-qPCR (**G**) and western blot (**H**) showed higher expression of NSUN2 in OS cell lines. The result of RT-qPCR was statistical analyzed according to the data of three independent experiments.

In order to evaluate the expression profile of NSUN2 in human OS, we analyzed the data from the GEO database (GSE126209: 11 normal and 11 OS tissues; GSE99671: 18 normal and 18 OS tissues), and found NSUN2 was highly expressed in OS tissues (Fig. 1A, B). Then, we analyzed the transcriptome data and the corresponding survival information in TARGET-OS from UCSC-XENA (http://xena.ucsc.edu/), and found higher expression of NSUN2 predicted poorer prognosis in OS patients (Fig. 1C). Moreover, we employed reverse transcription qPCR (RT–qPCR), immunohistochemistry (IHC) and western blot assays to detect the expression of NSUN2 in the normal and OS tumor tissues we collected from Wuhan University Zhongnan Hospital and the Affiliated Tumor Hospital of Zhengzhou University. The results of RT–qPCR (Fig. 1D), IHC (Fig. 1E), and western blot (Fig. 1F) indicated the expression of NSUN2 was higher in OS tissues. According to the NSUN2 IHC scores, 23 tumor tissues were identified as “high” (47.92%), 22 tumor tissues were identified as “medium” (45.83%), 3 tumor tissues were identified as “low” (6.25%); 5 normal tissues were identified as “high” (10.41%), 11 normal tissues were identified as “medium” (22.92%), and 32 normal tissues were identified as “low” (66.67%). The statistical graph of these results was shown in Fig. 1E. Furthermore, the higher expression of NSUN2 was also detected in OS cell lines (143b, MG63, U2) compared with that in bone mesenchymal stem cells (BMSCs) and human osteoblast (hFOB1.19) (Fig. 1G, H). Collectively, all these results imply that NSUN2 is upregulated in OS and higher expression of NSUN2 is correlated with poorer prognosis.

### NSUN2 deficiency significantly represses OS progression in vitro

**Fig. 2:**
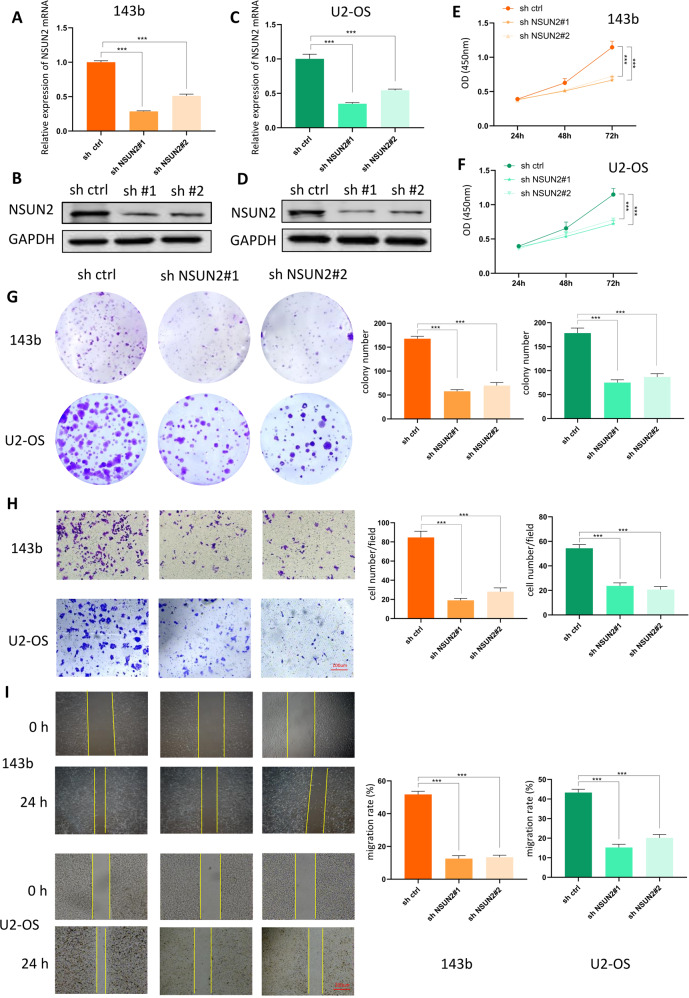
NSUN2 deficiency significantly represses OS progression in vitro. **A**, **B** The results of RT-qPCR (**A**) and western blot (**B**) showed the expression of NSUN2 in 143b cells. **C**, **D** The results of RT-qPCR (**C**) and western blot (**D**) showed the expression of NSUN2 in U2 cells. **E**–**G** The CCK-8 assay (**E**, 143b cells; **F**, U2 cells) and colony formation assay (**G**) showed that proliferation of OS cells decreased when NSUN2 was knocked down. **H** Transwell assay indicated that the invasion ability of OS cells decreased when the expression of NSUN2 decreased. **I** The results of the wound-healing assay confirmed that the cells in the sh-NSUN2 group had a lower migration ability. Statistical analysis was performed according to the data of three independent experiments.

To clarify the functional role of NSUN2 in OS progression, we chose 143b and U2 cell lines for further study. We used sh-NSUN2#1 and sh-NSUN2#2 to generate stable NSUN2-knockdown OS cell lines and then measured the proliferation, invasion, and migration abilities of these cells. First, we employed RT-qPCR and western blot to evaluate the expression of NSUN2 in the stably transfected 143b and U2 cells. The results of RT-qPCR and western blot showed sh-NSUN2#1 and sh-NSUN2#2 significantly knocked down the expression of NSUN2 in 143b (Fig. 2A, B) and U2 cells (Fig. 2C, D). As is indicated by Cell Counting Kit-8 (CCK-8) and colony formation assays, NSUN2 deficiency attenuated the proliferation ability of 143b cells and U2 cells (Fig. 2E–G). To measure the invasion and migration abilities of OS cells, we carried out wound-healing and transwell assays. The results confirmed that the invasion ability (Fig. 2H) and the migration ability (Fig. 2I) of 143b and U2 cells decreased when NSUN2 was knocked down. In general, these results suggest that knocking down NSUN2 expression negatively affects the progression of OS in vitro.

Furthermore, we overexpressed NSUN2 in 143b and U2 cells to observe the effect of NSUN2 overexpression on OS progression in vitro. The overexpression efficiency in 143b cells (Fig. S1A, B) and U2 cells (Fig. S1C, D) was tested by RT-qPCR and western blot. The results of CCK-8 assay, colony assay, transwell assay, and wound-healing assay together proved that NSUN2 overexpression had a promoting effect on the progression of OS in vitro (Fig. S1E–I).

### NSUN2 deficiency represses the proliferation of OS cells in vivo

**Fig. 3:**
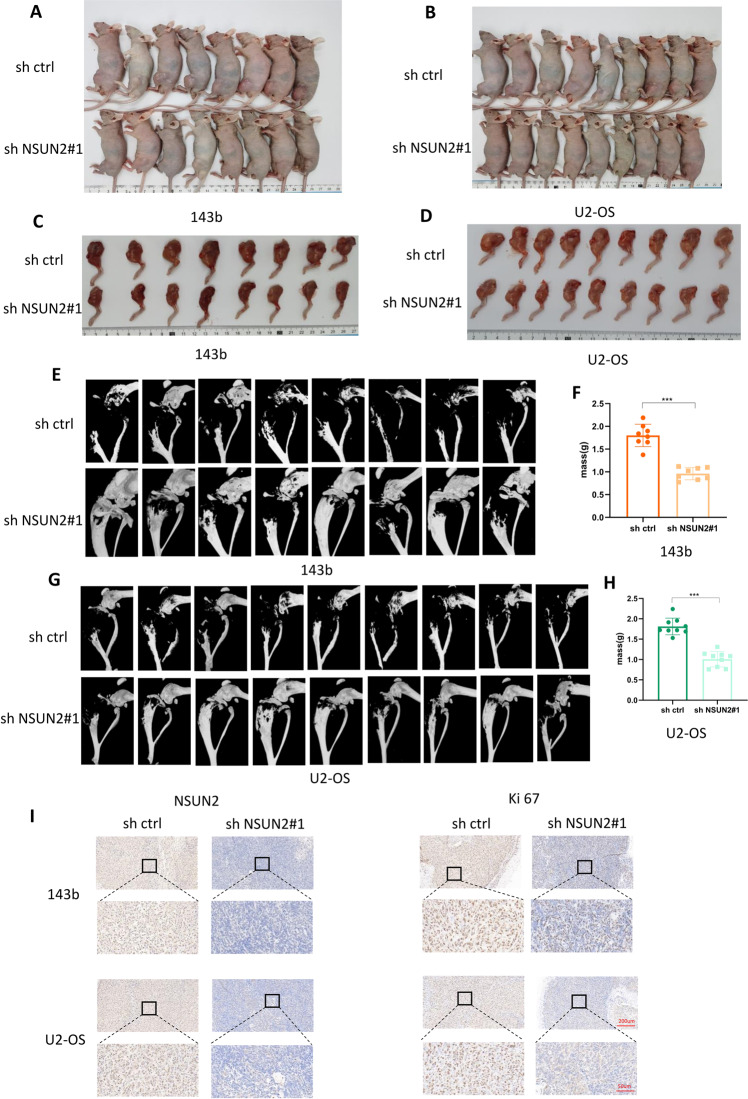
NSUN2 deficiency represses the proliferation of OS cells in vivo. **A**, **B** Tumor xenograft models were constructed with stable 143b and U2-OS cells (**A** for 143b cells, *n* = 8; **B** for U2-OS cells, *n* = 9). **C**, **D** Left legs of nude mice in the sh-NSUN2#1 group and the sh-ctrl group. **E**, **G** 3D images of micro-CT scans for the tumors. **F**, **H** Final weights of the tumors. **I** IHC of NSUN2 and Ki-67 in tumors.

We generated tumor xenografts in nude mice with 143b cells and U2 cells stably transfected with sh-NSUN2#1 or sh-ctrl. We inoculated stably transfected OS cells into the upper segment of the left tibia in nude mice, and then we observed the tumor size for three weeks. Next, we sacrificed all the nude mice and collected the left leg for microcomputed tomography (micro-CT) scanning. In addition, we collected and weighed all the tumors. As is expected, the final size of tumors in the sh-NSUN2#1 group was smaller than that in the sh-ctrl group (Fig. 3A–D). Furthermore, the results of micro-CT scanning revealed that the bone destruction in the left leg was less severe and that the tumor volume was smaller in the sh-NSUN2#1 group (Fig. 3E, G). In addition, the weight of tumors was less in the sh-NSUN2#1 group (Fig. 3F, H). The IHC results confirmed that the expression of NUSN2 and Ki-67 was decreased in tumors in the sh-NSUN2#1 group (Fig. 3I). All the above results indicate that NSUN2 deficiency inhibits the proliferation of OS cells in vivo.

### FABP5 is the potential target of NSUN2

**Fig. 4:**
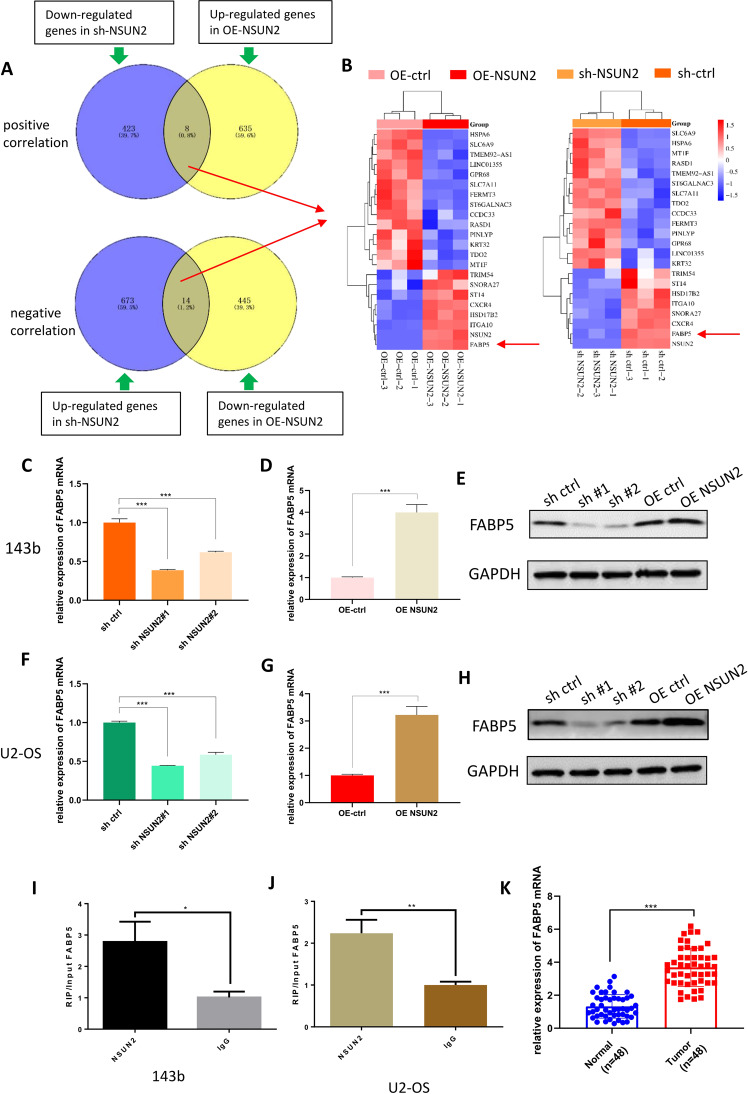
FABP5 is the potential target of NSUN2. **A** Venn diagram of RNA-seq showing the genes whose expression level was positively or negatively correlated with the expression level of NSUN2. The sh-NSUN2 group means the cells were stably transfected with sh-NSUN2#1. **B** The heatmaps made by the genes obtained from (**A**). **C**, **D** The results of RT-qPCR showed the expression level of FABP5 mRNA when NSUN2 was knocked down or overexpressed in 143b cells. **E** The results of western blot showed the expression level of FABP5 protein when NSUN2 was knocked down or overexpressed in 143b cells. **F**, **G** The results of RT-qPCR showed the expression level of FABP5 mRNA when NSUN2 was knocked down or overexpressed in U2 cells. **H** The results of western blot showed the expression level of FABP5 protein when NSUN2 was knocked down or overexpressed in U2 cells. **I**, **J** The results of RIP and RT-qPCR in 143b and U2 cells. IgG was used as a negative control to preclude nonspecific binding. **K** The result of RT-qPCR showed FABP5 was highly expressed in OS tissues. The result of RT-qPCR was statistical analyzed according to the data of three independent experiments.

To identify the downstream target of NSUN2 in OS cells, we performed RNA-seq with 143b cells stably transfected with sh-NSUN2#1 or OE-NSUN2 (sh-NSUN2 VS sh-ctrl; OE-NSUN2 VS OE-ctrl). The RNA-seq was completed by RiboBio Co., Ltd (Guangzhou, China). The result of similarity analysis was shown in Fig. S2A, and the volcano plots are shown in Fig. S2B (sh-NSUN2 VS sh-ctrl) and Fig. S2C (OE-NSUN2 VS OE-ctrl). We focused on those genes whose expression level was positively or negatively correlated with that of NSUN2 (Fig. 4A), and made two heatmaps containing all these genes (Fig. 4B). From the heatmaps, we concluded that the expression of FABP5 was most significantly changed in both “sh-NSUN2 VS sh-ctrl” and “OE-NSUN2 VS OE ctrl”. Therefore, we hypothesized that FABP5 was the potential target of NSUN2 in OS cells, and NSUN2 promoted the progression of OS by regulating FABP5 expression. To test our hypothesis, we used RT-qPCR to detect the expression level of FABP5 mRNA in stably transfected 143b and U2 cells, and found the expression level of FABP5 mRNA decreased significantly when NSUN2 was knocked down (Fig. 4C, F) and increased when NSUN2 was overexpressed (Fig. 4D, G). Furthermore, the results of western blot also confirmed the expression level of FABP5 protein decreased when NSUN2 was knocked down and increased when NSUN2 was overexpressed (Fig. 4E, H).

To find out whether NSUN2 affects FABP5 expression through direct or indirect action, we performed RNA immunoprecipitation (RIP) assay to detect whether NSUN2 protein could bind to FABP5 mRNA. We used magnetic beads and antibodies against NSUN2 to isolate RNA that can interact with NSUN2 protein from 143b and U2 cells, and detected these RNA with RT-qPCR. The results showed FABP5 mRNA was included in the RNA we isolated from 143b and U2 cells (Fig. 4I, J), suggesting that NSUN2 protein could directly bind to FABP5 mRNA. All the above results together indicated NSUN2 protein can directly target on FABP5 mRNA and affect the expression level of FABP5.

To further test our conjecture, we detected the expression of FABP5 mRNA in OS tissues and normal tissues we collected with RT-qPCR, and the result confirmed FABP5 was also highly expressed in OS tissues (Fig. 4K). This result further supported our conjecture that FABP5 was the potential target of NSUN2 in OS cells.

### NSUN2 promotes the stability of FABP5 mRNA via m^5^C

**Fig. 5:**
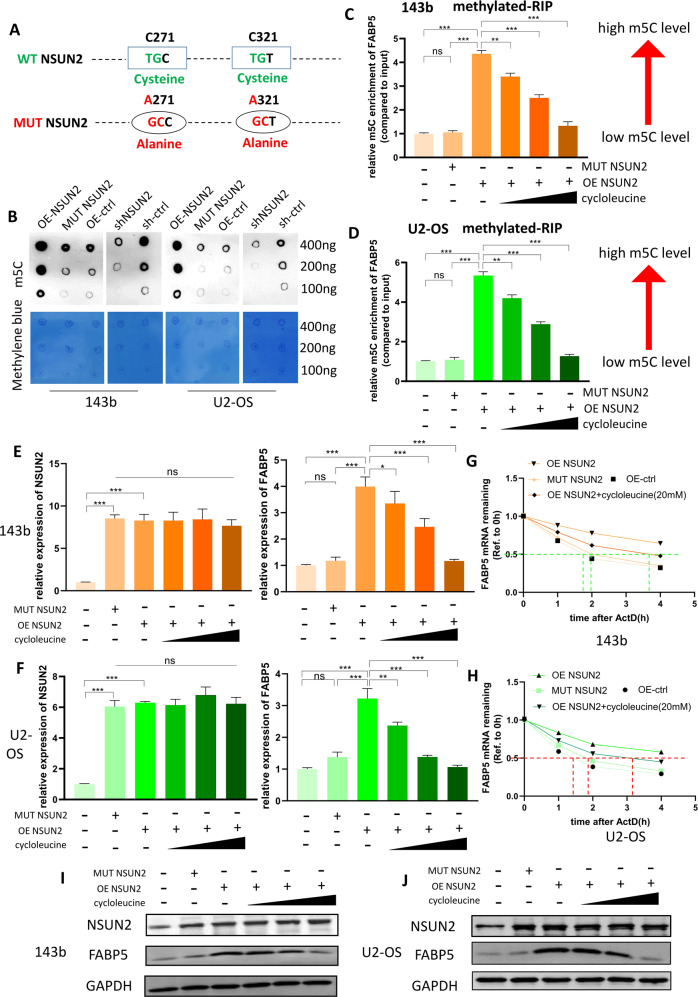
NSUN2 promotes the stability of FABP5 mRNA via m^5^C. **A** The schematic diagram showed that two cysteines of NSUN2 (C271 and C321) were mutated into alanine. The sh-NSUN2 group means the cells were stably transfected with sh-NSUN2#1. **B** An m^5^C dot blot assay was used to detect the m^5^C levels of mRNA extracted from stably transfected 143b and U2-OS cells. RNA was serially diluted and loaded equally at 400 ng, 200 ng, and 100 ng. Methylene blue staining (below) was used to detect the amount of RNA loaded, while the intensity of dot immunoblotting (above) represented the level of m^5^C modification. **C**, **D** The results of methylated-RIP for 143b and U2 cells. The relative m^5^C enrichment of FABP5 mRNA for each group was normalized to the Input. The low concentration of cycloleucine was 10 mM, the middle was 20 mM, and the high was 40 mM. **E**, **F** The results of RT–qPCR showing the expression of NSUN2 mRNA and FABP5 mRNA in 143b and U2 cells. **G**, **H** The curve of FABP5 mRNA remaining versus time after ActD treatment in 143b and U2 cells. **I**, **J** The results of western blot showing the expression of FABP5 protein and NSUN2 protein. The result of RT-qPCR was statistical analyzed according to the data of three independent experiments.

To further verify whether NSUN2 regulates FABP5 expression via m^5^C, we produced an NSUN2 overexpression plasmid with mutation sites. Previous reseaches have proved that NSUN2 mainly relies on the two cysteines at C271 and C321 sites to play the role of methyltransferase. The cysteine at C321 site forms covalent bonds with the pyrimidine ring of cytosine to catalyze the methylation of cytosine, while The cysteine at C271 site mediates the release of methylated RNA [19, 20]. Therefore, we mutated the “TG” into “GC” at the C271 site of NSUN2, so that the original sequence “TGC” was mutated into “GCC”. In this way, we mutated the cysteine into alanine at the C271 site on NSUN2. Using the same method, we also mutated cysteine into alanine at C321 site on NSUN2 (Fig. 5A). Then, we transfected the mutant NSUN2 plasmid into 143b and U2 cells, and detected the m^5^C level of all the stably transfected cells with dot blot assay. We found sh-NSUN2 led to a lower m^5^C level while overexpression of NSUN2 led to a high m^5^C level (Fig. 5B). However, the cells transferred into the mutant NSUN2 plasmid did not show any change in m^5^C level (Fig. 5B).

To confirm whether NSUN2 can regulate the m^5^C level of FABP5 mRNA, we carried out methylated-RIP assay with stably transfected 143b and U2 cells. We used magnetic beads and m^5^C antibodies to isolate RNA, and analyzed these RNA by RT-qPCR. Higher content of an mRNA in the isolated RNA means higher m^5^C level of the mRNA. In order to make our research more convincing, we treated OS cells overexpression NSUN2 with cycloleucine, which could reduce mRNA methylation and has wildly used in the reseaches of RNA methylation. As a competitive inhibitor of methionine adenosyltransferase 2 (MAT II), cycloleucine can reduce mRNA methylation by depleting the source of the methyl group. In theory, cycloleucine can reduce the level of all types of methylation, including m^6^A, m^5^C and so on, because it can reduce the source of the methyl group. We then performed m^6^A MeRIP-qPCR and found that FABP5 mRNA was not regulated by m^6^A modification in OS cells (Fig. S2D). Therefore, in this study, the cycloleucine we used only reduced the m^5^C level of FABP5 mRNA. The results of m^5^C methylated-RIP showed that overexpression of NSUN2 could promote the m^5^C level of FABP5 mRNA in 143b and U2 cells, but mutant NSUN2 could not. Cycloleucine could reduce the m^5^C level of FABP5 mRNA in 143b and U2 cells in a concentration-dependent manner (Fig. 5C, D). Then, we detected the expression of NSUN2 and FABP5 in 143b and U2 cells with RT-qPCR. The results confirmed that MUT-NSUN2 could promote the expression of NSUN2, but could not promote the experssion of FABP5. Cycloleucine could reduce the expression of FABP5 without reducing the expression of NSUN2 (Fig. 5E, F). Collectively, higher m^5^C level led to higher expression of FABP5 mRNA.

To figure out how NSUN2-mediated m^5^C regulates the expression of FABP5 mRNA. We detected the stability of FABP5 mRNA in 143b and U2 cells with actinomycin D (ActD, 5 µg/ml). After ActD treatment for 0, 1, 2, or 4 h, RNA was extracted and analyzed by RT–qPCR. The stability of FABP5 mRNA was determined by the degradation rate (normalized to the expression at 0 h). As is shown in Fig. 5G and Fig. 5H, the higher the m^5^C level of FABP5 mRNA, the longer it takes to decay to 50%, and the better the stability. In other words, NSUN2 could promote the stability of FABP5 mRNA in OS cells via m^5^C. Furthermore, we carried out western blot to detect the expression of FABP5 protein in 143b and U2 cells, and found that the higher the m^5^C level of FABP5 mRNA, the higher the expression of FABP5 protein (Fig. 5I, J).

YBX1, as an important m^5^C reader, has been proved to maintain mRNA stability by lots of studies [10, 13, 14]. To verify whether YBX1 was the functional m^5^C reader in OS cells, we performed RIP assay with YBX1 antibody in 143b and U2 cells, and the result of RT-qPCR showed YBX1 could bind to FABP5 mRNA, indicating YBX1 might be the m^5^C reader (Fig. S3A, B). Then, further research proved that sh-YBX1 could decrease the expression of FABP5 by down-regulating the stability of FABP5 mRNA in 143b and U2 cells (Fig. S3C–J). The results together proved that YBX1 could participate in regulating the stability of FABP5 mRNA as a functional reader.

Taking all the above results into consideration, we could draw such a conclusion that NSUN2 could promote the expression of FABP5 by up-regulating the stability of FABP5 mRNA in a m^5^C dependent manner in OS cells.

### NSUN2 promotes fatty acid metabolism in OS cells

**Fig. 6:**
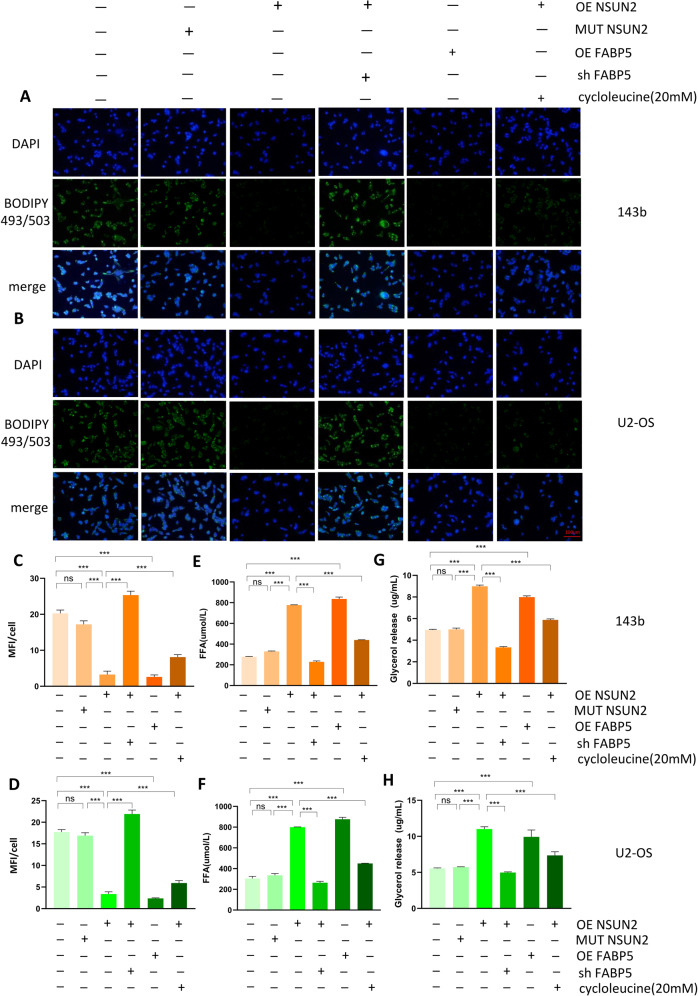
NSUN2 promotes fatty acid metabolism in OS cells. **A**, **B** The level of neutral lipids in OS cells was detected by BODIPY 493/503. **C**, **D** Quantification and statistics of the mean fluorescence intensity (MFI) for stably transfected OS cells. **E**, **F** The level of FFAs in stably transfected OS cells. **G**, **H** The level of glycerol in stably transfected OS cells. The sh-NSUN2 group means the cells were stably transfected with sh-NSUN2#1. Statistical analysis was performed according to the data of three independent experiments.

As is known to all, FABP5 plays a very important role in fatty acid metabolism, and we have confirmed that NSUN2 can up-regulate the expression of FABP5 by stabilizing its mRNA in OS cells. To evaluate whether NSUN2 could promote fatty acid metabolism in OS cells, we first examined the level of neutral lipids with BODIPY 493/503 in stably transfected 143b and U2 cells. To make our study more rigorous, we treated the stably transfected OS cells with fatty acid oxidation inhibitor, Etomoxir (1 mmol/L, MedChemExpress, USA). The content of neutral lipids was evaluated by detecting the mean fluorescence intensity (MFI) of OS cells. The results showed sh-NSUN2 led to the accumulation of neutral lipids, while NSUN2 overexpression resulted in the shrinkage of neutral lipids in OS cells. The sh-NSUN2 group means the cells were stably transfected with sh-NSUN2#1. Similar to NSUN2, FABP5 overexpression can also lead to the reduction of neutral lipids, and knockdown can increase the accumulation of neutral lipids in OS cells. Furthermore, MUT-NSUN2 failed to change the content of neutral lipids in OS cells, while Etomoxir increased the content of neutral lipids in OS cells. Compared with NSUN2 overexpression, cycloleucine could reduce FABP5 expression by reducing m^5^C level of FABP5 mRNA, so as to affect the content of neutral lipids in OS cells (Fig. 6A–D, Fig. S4A, B). In general, in contrast to the effect of Etomoxir, higher FABP5 expression predicted lower level of neutral lipids in OS cells. Moreover, we detected the content of free fatty acids (FFAs) and glycerol to evaluate lipolytic activity in OS cells. The results suggested that both OE-NSUN2 and OE-FABP5 could promote the level of FFAs and glycerol in OS cells, while both sh-NSUN2 and sh-FABP5 could reduce the level of FFAs and glycerol in OS cells. As is expected, MUT-NSUN2 failed to change the content of FFAs and glycerol in OS cells, while Etomoxir decreased the content of FFAs and glycerol in OS cells (Fig. 6E–H, Fig. S4C–F). In other words, higher FABP5 expression predicted higher lipolytic activity in OS cells. Taking all the above results into consideration, NSUN2 promotes lipolysis of neutral lipids by up-regulating the expression of FABP5 in OS cells.

### Both fatty acid oxidation inhibitor and FABP5 deficiency can counterbalance the positive effect of NSUN2 on OS progression

**Fig. 7:**
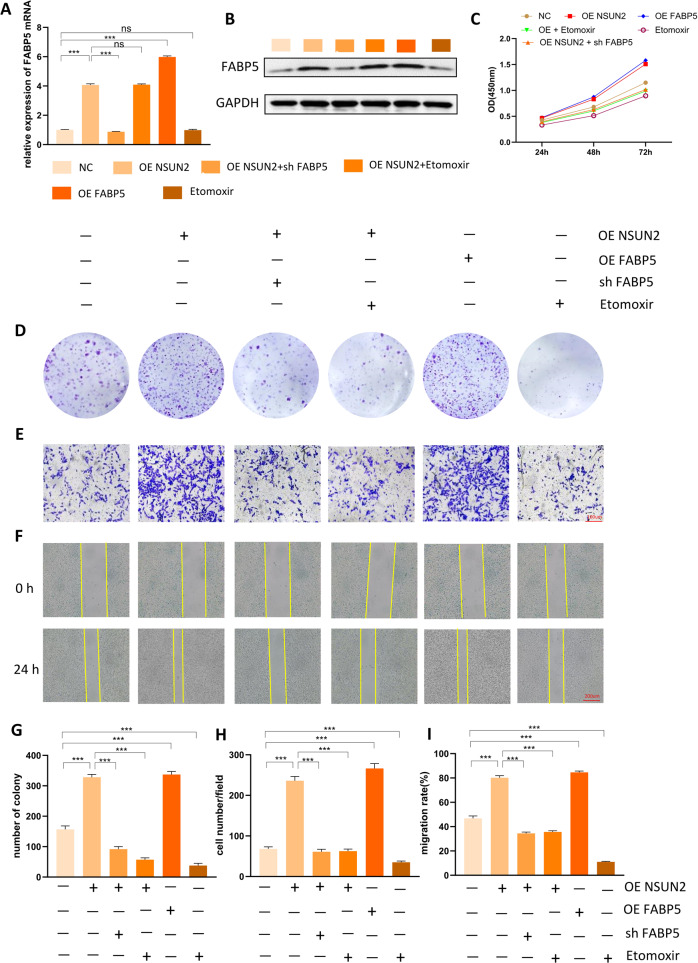
Both fatty acid oxidation inhibitor and FABP5 deficiency can counterbalance the positive effect of NSUN2 on OS progression. **A**, **B** The expression level of FABP5 mRNA (**A**) and protein (**B**) in 143b cells of each group. **C** The results of CCK-8 assay. **D** The results of colony formation assay. **E** The results of the transwell assay. **F** The results of the wound-healing assay. **G** Quantification and statistics of colony formation assay. **H** Quantification and statistics of transwell assay. **I** Quantification and statistics of wound-healing assay. Statistical analysis was performed according to the data of three independent experiments.

To further confirm whether NSUN2 facilitates the progression of OS by promoting the expression of FABP5 and fatty acid metabolism, we performed functional rescue assays with 143b cells and U2 cells. The results of 143b cells were displayed in Fig. 7 and the results of U2 cells were displayed in Fig. S5. We first examined the expression level of FABP5 mRNA (Fig. 7A; Fig. S5A) and protein (Fig. 7B; Fig. S5B) in 143b and U2 cells of each group. As is indicated by the results of CCK-8 (Fig. 7C; Fig. S5C) and colony formation assays (Fig. 7D, G; Fig. S5D, G), NSUN2 overexpression had a promoting effect on the proliferation of 143b cells, but this promoting effect could be counterbalanced by Etomoxir or the loss of FABP5. Moreover, FABP5 overexpression had the similar promoting effect on the proliferation of 143b cells as NSUN2 overexpression. In addition, Etomoxir could reduce the proliferation of 143b cells. The similar results were gotten in transwell and wound-healing assays. NSUN2 overexpression had a positive effect on the migration and invasion of 143b cells, but this positive effect was offset by FABP5 deficiency or Etomoxir. FABP5 overexpression was also found to have a positive effect on the migration and invasion of 143b cells, while Etomoxir had a negative effect (Fig. 7E, F, H, I; Fig. S5E, F, H, I).

In addition, we used animal models to further validate the functional role of NSUN2-FABP5 axis in OS progression, and the results were displayed in Fig. S6. As is shown in Fig. S6A–H, NSUN2 overexpression could promote the proliferation of OS cells in vivo, while sh-FABP5 could attenuate such promoting effect. The results of IHC (Fig. 6I, J) also proved that NSUN2 overexpression could improve the expression of Ki-67, while sh-FABP5 could offset such promoting effect.

Collectively, all the above results prove that NSUN2 facilitates the progression of OS by promoting the expression of FABP5 and fatty acid metabolism, and this promoting effect can be counterbalanced by loss of FABP5 or fatty acid oxidation inhibitor, Etomoxir.

## Discussion

Accumulating evidence has demonstrated that posttranscriptional modifications, including m^6^A and m^5^C, play critical roles in the progression of many cancers [21]. It has been reported that m^6^A mediated by WTAP plays a crucial role in the progression of OS [22]. However, the functions of m^5^C regulators in OS progression remain unclear. In this study, we focused on the role and underlying mechanism of NSUN2 and NSUN2-mediated m^5^C modification in OS progression. Functionally, NSUN2 deficiency negatively affected the progression of OS, while NSUN2 overexpression facilitated the progression of OS. Mechanistically, RNA-seq, RIP, and methylated RIP were employed to identify FABP5 as the target of NSUN2. Further study confirmed that NSUN2 positively affected the progression of OS by stabilizing FABP5 mRNA via m^5^C and promoting fatty acid metabolism. Our work is the first to reveal the role of NSUN2-mediated m^5^C modification in the progression of OS and clarify its mechanism.

NSUN2 was considered as a tRNA methyltransferase, and many studies have focused on its function in promoting tRNA stability and protein synthesis [23, 24]. Over the last few years, NSUN2 and m^5^C modification have attracted increasing attention, and an increasing number of researchers have begun to study their functional roles in mRNA and the progression of many diseases. For example, several studies have shown that m^5^C modification plays important roles in the progression of breast cancer [25], lung adenocarcinoma [26], and lung squamous cell carcinoma [27] by regulating the tumor microenvironment. In addition, several studies have reported that m^5^C regulators may predict the prognoses of patients with head and neck squamous cell carcinoma [28], pancreatic adenocarcinoma [29], hepatocellular carcinoma [30], or ovarian cancer [31]. NSUN2 has also been reported to stabilize HDGF mRNA via m^5^C and to promote the progression of bladder cancer [14]. Apart from cancers, NSUN2-mediated m^5^C can regulate adipogenesis by promoting CDKN1A mRNA export and translation [32]. Overall, these previous studies have identified NSUN2 as an oncogene in many cancers, whose main function is to stabilize target mRNA or to promote export and translation of the target mRNA. Our study got a similar conclusion that NSUN2 plays a promoting role in OS progression. Next, we shed light on the downstream mRNAs modified by NSUN2. The results of RNA-seq, RIP, and mehylated RIP collectively identified FABP5 as the potential target. Subsequently, the results of RT–qPCR, western blot, and RNA stability assays confirmed that NSUN2 facilitates the progression of OS by stabilizing FABP5 mRNA via m^5^C.

The regulatory effects of RNA m^5^C modification are usually interpreted by their reader protein, and YBX1 is the most well-known reader protein that can maintain mRNA stability. In this study, we identified YBX1 as the m^5^C reading protein and found FABP5 could participate in the regulation of the stability of FABP5 mRNA. Similar conclusions have been reported in previous literature. For example, YBX1 has been found to be able to work with NSUN2 to stabilize HDGF mRNA to promote the progression of bladder cancer [14].

FABP5, which belongs to the family of FABPs, has a high affinity for fatty acids and is the key protein for fatty acid metabolism [33]. FABP5 participates in the processes of extracellular fatty acid transport and absorption, transfer of fatty acids from the cytoplasm to the nucleus, and activation of nuclear receptors that regulate the expression of NF-κB and other genes [34–36]. In this study, we found that NSUN2 increased the expression of FABP5 in OS cells and that fatty acid metabolism in OS cells could be upregulated by NSUN2. We evaluated the level of fatty acid metabolism by measuring the levels of neutral lipids, FFAs, and glycerol in OS cells. Moreover, our work proved that both loss of FABP5 and fatty acid oxidation inhibitor could offset the positive effect of NSUN2 on OS progression. Given all the findings, we conclude that NSUN2 facilitates OS progression by promoting FABP5 mRNA stability via m^5^C and fatty acid metabolism.

To determine whether NSUN2 promotes the progression of OS by up-regulating the expression of FABP5, we overexpressed FABP5 and found FABP5 overexpression had the similar promoting effect for OS progression as NSUN2 overexpression (Fig. 7). In addition, the result of RIP also confirmed that NSUN2 protein can directly bind to FABP5 mRNA. Actually, a lot of studies have proved that FABP5 has a promoting effect in many cancers. For instance, FABP5 has been reported to promote tumor progression by mediating fatty acid metabolism and stabilizing PI3K/AKT/mTOR signaling in lung adenocarcinoma [37]. FABP5 has also been proven to enhance the malignancy of lower-grade gliomas via canonical activation of NF-κB signaling [38]. Moreover, silencing FABP5 has been found to decrease the proliferation of prostate cancer cells in vitro and in vivo [39]. FABP5 has been found to promote lymph node metastasis in cervical cancer by reprogramming fatty acid metabolism [33]. In addition, fatty acid metabolism regulated by FABP5 has been also confirmed to play crucial roles in the progression of many cancers. For example, fatty acid metabolism provides structural phospholipids such as cardiolipin, triglyceride, and glycerin for incorporation into malignant tumor cell membranes [40–42]. Specifically, Frankie reported that FASN can promote the growth of OS cells by promoting the synthesis of fatty acids [43]. In addition, fatty acid metabolism has been reported to be associated with the progression of prostate cancer [44], bladder cancer [45], breast cancer [46], liver cancer [47], and other cancers. The conclusion of our study is similar to the conclusions of these reported studies. We found that FABP5 played an important role in the progression of OS, and was regulated by NSUN2 via m^5^C in OS.

In summary, our study can draw the conclusion that NSUN2 up-regulates the expression of FABP5 by improving the stability of FABP5 mRNA via m^5^C, so as to promote fatty acid metabolism in OS cells, and finally plays the role in promoting the progression of OS.

## Materials and methods

### Bioinformatics analysis

In this study, we downloaded and analyzed the data of GSE126209 and GSE99671 from GEO database (www.ncbi.nlm.nih.gov). In addition, we downloaded and analyzed the transcriptome data and the corresponding survival information in TARGET-OS from UCSC-XENA (http://xena.ucsc.edu/). Gencode platform (https://www.gencodegenes.org/) was used and Transcripts Per Kilobase Million (TPM) normalization was performed in TARGET-OS.

### Patients and clinical samples

This research was approved by the Institutional Ethics Committee of Wuhan University Zhongnan Hospital (No. 2021050) and the Affiliated Tumor Hospital of Zhengzhou University (2021-KY-0148-001). Forty-eight patients from Wuhan University Zhongnan Hospital and the Affiliated Tumor Hospital of Zhengzhou University were included in this study, and we obtained OS tissues and the corresponding peritumoral tissues from these patients. Total RNA and proteins were isolated from some of the tissues for RT–qPCR and western blot assays. Another portion of the tissues was used for IHC performed by Servicebio (Wuhan, China). Written informed consent was acquired from all the patients. The IHC scores of all the tumor and normal tissues were decided according to the percentage of positive‐staining cells (high, >75%; medium, 25% ~75%; low, <25%). The IHC scores were evaluated by three pathologists independently.

### Cell culture

The human OS cell lines 143b, MG63, U2-OS, and BMSCs were cultured at 37 °C and the osteoblast cell line hFOB 1.19 was cultured at 34 °C in a 5% CO_2_ incubator (Thermo Fisher, USA). The 143b cells were cultured in RPMI-1640 (Gibco, USA), the U2-OS cells were cultured in McCoy’s 5A medium (Gibco, USA), the hFOB 1.19 cells were cultured in DMEM (Gibco, USA), and the BMSCs were cultured in αMEM (Gibco, USA). All cells were routinely supplemented with 10% fetal bovine serum (FBS) (Gibco, Scoresby, Australia) and 1% penicillin and streptomycin.

### Cell transfection

Lentiviruses with puromycin tags were purchased from Hanbio (Shanghai, China). Using polybrene (Hanbio, Shanghai China), OS cells were stably transfected with the NSUN2-knockdown lentivirus (sh-NSUN2), the NSUN2 overexpression lentivirus (OE-NSUN2), the FABP5-knockdown lentivirus (sh-FABP5), FABP5 overexpression lentivirus (OE-FABP5), YBX1-knockdown lentivirus (sh-YBX1), or the negative control (sh ctrl, OE-ctrl). The detailed lentivirus sequence information has been listed in Table 1. Next, the transfected OS cells were selected by puromycin (5 μg/ml) for further studies. Western blot and RT–qPCR were used to detect the transfection efficiencies. A mutant plasmid of NSUN2 was synthesized by Hanbio (Shanghai, China) and transfected into 143b and U2-OS cells with Lipofectamine 3000 (Hanbio, Shanghai China).

**Table 1:** Lentivirus sequence information.

sh NSUN2#1	CGGCCTCATCATAAGATCTTAGATA
sh NSUN2#2	CAAAGGGAAGCATCGTGCTGAAGTA
OE NSUN2	F: actagaggatctatttccggtgaattcGCCACCatggggcggcggtcg
	R: cagatccttactagtatcgatggatcctcaTTTTGGAGGATGGTCGCCA
sh FABP5	CACAGCTGATGGCAGAAAA
OE FABP5	F: CGGGATCCGCCACCATGGCCACAGTTCAGCAGCT
	R: ATAAGAATGCGGCCGCTTATTCTACTTTTTCATAGA
sh YBX1	GTTCAATGTAAGGAACGGATA

### Reverse transcription qPCR (RT–qPCR)

Total RNA was extracted from cells and tissues using TRIzol reagent (Invitrogen, USA). The RNA was reverse-transcribed into cDNA by reverse transcriptase (Vazyme Biotech Co., Ltd.) and then subjected to an RT–qPCR assay using ChamQ Universal SYBR qPCR Master Mix (Vazyme Biotech Co., Ltd.). All quantified values were normalized to the endogenous GAPDH expression levels. The sequences of the primers used for RT–qPCR are listed in Table 2.

**Table 2:** Primers sequences used for RT–qPCR.

NSUN2	Sense	GTTTGACTGTGCTTTCCGGC
	Antisense	CTTCAGCACGATGCTTCCCT
FABP5	Sense	AGGAGCTAGGAGTGGGAATAGC
	Antisense	CTGAGTTTTTCTGCCATCAGCT
GAPDH	Sense	CAAATTCCATGGCACCGTCA
	Antisense	GACTCCACGACGTACTCAGC

### RNA m^5^C dot blot assay

After total RNA was extracted from 143b and U2-OS cells, mRNA was isolated from the total RNA and denatured at 65 °C for 5 min. Different amounts of mRNA (100, 200, or 400 ng) were loaded onto an Amersham Hybond N + membrane (GE Healthcare). After being crosslinked with ultraviolet (UV) light for 5 min, the membrane was stained with 0.02% methylene blue (Sangon Biotech, China). Next, the membrane was blocked with 5% nonfat dried milk in phosphate-buffered saline (PBS) with Tween-20 (PBST), and the membrane was then incubated with an anti-m^5^C antibody (1:1000, Abcam, ab214727) overnight at 4 °C. Then, after incubation with the secondary antibody, the membrane was visualized with an imaging system.

### Cell Counting Kit-8 (CCK-8) and colony formation assay

A CCK-8 assay was performed to measure the proliferation ability of stably transfected OS cells. Cells were plated at a density of 1000 cells/well in 96-well plates. At 24, 48, and 72 h after plating, 100 µl of complete medium, including 10% CCK-8 working solution, was added to each well, and the cells were incubated for 2 h. The optical density (OD) was measured at 450 nm using a microplate reader.

For the colony formation assay, stably transfected OS cells were plated in 6-well plates at a density of 1000 cells/well and cultured for 2 weeks. Then, the colonies were photographed and counted after 4% paraformaldehyde (Solarbio) and 1% crystal violet (Solarbio) were employed to fix and stain the OS cells.

### Transwell and migration assays

Transwell assays were applied to measure the invasion ability of stably transfected OS cells, while wound-healing assays were employed to evaluate the migration ability of these OS cells. For the transwell assay, the upper chambers were covered with Matrigel (Corning, NY, USA). Cells were plated in the upper chamber at a density of 2 × 10^4^ cells/well with culture medium containing 1% FBS, while the bottom chambers contained culture medium with 20% FBS. After 24 h of incubation, the cells invading the lower surfaces of the chambers were fixed in 4% paraformaldehyde (Solarbio), stained with 1% crystal violet (Solarbio), and quantified by counting 4 random fields of view.

For the wound-healing assay, stably transfected OS cells were plated in 6-well plates, and an artificial wound was created using a 1000 μL pipette tip when the cell confluence reached 90%. The cells were then cultured with medium containing 1% FBS for 24 h. Images were taken to record the width of the wound at 0 and 24 h. Cell migration ability was evaluated by comparing the wound widths of the different groups at 0 and 24 h.

### RNA immunoprecipitation (RIP)

The OS cells were crosslinked with 0.3% formaldehyde to strengthen the binding of RNA and protein. Magnetic beads were mixed with 5 µg of anti-NSUN2 (Abcam, ab259941) or anti-rabbit IgG (Abcam, ab172730), the treated magnetic beads were added to OS cell lysates, and the mixtures were incubated at 4 °C for 12 h. In this way, magnetic bead–antibody–protein–RNA complexes were formed. Next, proteinase K digestion buffer was added to digest the complexes for 45 min at 55 °C. After that, buffer (phenol:chloroform:isoamyl alcohol = 125:24:1) was employed to extract the RNA. The extracted RNA was subsequently analyzed by RT–qPCR. IgG was used as a negative control to preclude nonspecific binding.

### Methylated RNA immunoprecipitation (methylated RIP)

The process of RNA enrichment for methylated RIP is very similar to that for RIP. Briefly, approximately 150 μg of total RNA isolated from stably transfected OS cells was immunoprecipitated with magnetic beads precoated with 10 μg of anti-m^5^C antibody (Abcam, ab214727), anti-m^6^A antibody (Abcam, ab208577), or anti-IgG (Abcam, ab172730). In this way, magnetic bead–antibody–RNA complexes were formed. After that, the complexes were digested by proteinase K digestion buffer so that the RNA combined with the antibody was eluted from the complex. Similarly, buffer (phenol:chloroform:isoamyl alcohol = 125:24:1) was employed to extract the RNA. Then, the extracted RNA was further analyzed by RT–qPCR. The sequences of the primers used for methylated RIP RT-qPCR were: 5′-3′, F: GCAGACCCCTCTCTGCAC; R: AAGCCTTTGCTGTCCACCAG. The relative m^5^C enrichment of mRNA was normalized to the Input.

### mRNA stability assay

To analyze mRNA stability, stably transfected OS cells were treated with actinomycin D (5 µg/ml). Then, the cells were collected, and total RNA was extracted using TRIzol reagent at different time points (0, 1, 2, and 4 h after ActD treatment). Reverse transcription was then performed, and mRNA levels were measured by RT–qPCR. The mRNA levels were normalized to the expression at 0 h

### Western blot

Total proteins extracted from OS cells and tissues were separated by SDS–PAGE and then transferred onto PVDF membranes. The membranes were incubated with primary antibodies (NSUN2, Abcam, ab259941; FABP5, Abcam, ab84028; YBX1, Abcam, ab76149; GAPDH, Abcam, ab8245) at 4 °C for 12 h and with secondary antibodies for 1 h. Then, visualization was performed using an Odyssey Infrared Imaging System (LI-COR Biosciences, USA). The ODs of the membrane blots were analyzed using ImageJ software.

### Quantification of neutral lipids, free fatty acids (FFAs), and glycerol

We used the fluorescent dye BODIPY 493/503 (Invitrogen) to detect the levels of neutral lipids in OS cells. After being fixed with 4% paraformaldehyde, OS cells were stained with BODIPY 493/503 (1 µg/mL) for 30 min at room temperature. DAPI (20 µL/mL) was then added as a nuclear counterstain, and the cells were incubated with DAPI for 15 min. The staining was visualized by fluorescence microscopy after the cells were washed with PBS three times. Neutral lipids were quantified with ImageJ software.

An FFA detection kit (Solarbio, China) was used to measure the levels of FFAs in OS cells. Briefly, FFAs were released from OS cells after the membranes of the OS cells were destroyed. Then, a standard curve was drawn on the basis of the standard sample according to the manufacturer’s protocols. The OD values of the OS cells were measured, and the levels of FFAs in the OS cells were calculated from the OD values we measured and the standard curve. A glycerol detection kit (Sigma, USA) was used to quantify the glycerol in OS cells according to the manufacturer’s protocols.

### Tumor xenograft model

The animal study was approved by the Ethics Committee of Zhongnan Hospital of Wuhan University (WQ20210015). This study was executed at the Animal Laboratory Center, Zhongnan Hospital of Wuhan University. Four-week-old male BALB/c nude mice were purchased from GemPharmatech (Jiangsu, China). Every group contains three or ten mice, and the mice will not be included for statistical analysis once the mice is dead or the tumor diameter is greater than 2 cm. Stably transfected OS cells were collected and then resuspended in PBS (approximately 10^7^/mL). Next, 100 µL of cells (containing approximately 10^6^ cells) was injected into the left upper tibia or the upper right back of each mouse. After three weeks, the mice were sacrificed, and the tumor tissues were collected. The tumor tissues were then weighed, measured, or scanned by micro-CT. The volume of subcutaneous tumors was calculated as “tumor volume (mm^3^) = length × width^2^ × 0.5”. IHC of the tumors was performed by Servicebio (Wuhan, China).

### Statistical analysis

All values in this study are presented as the means ± SDs from three independent experiments. Statistical significance was determined by one-way ANOVA or unpaired two-tailed Student’s t test. One-way ANOVA was used when more than two groups were compared and was followed by the Bonferroni multiple comparisons post hoc test. Statistical analysis was performed according to the data of three independent experiments with GraphPad Prism 8.0 (GraphPad, Inc., USA). *P* < 0.05 was considered to indicate statistical significance (*P < 0.05, **P < 0.01, ***P < 0.001, ****P < 0.0001).

## Supplementary information

Figure S1

Figure S2

Figure S3

Figure S4

Figure S5

Figure S6

supplementary figure legends

the original data of western blot

Author Contribution Statement

reproducibility checklist

editing certificate

## Data Availability

Authors can confirm that all relevant data are available on request from the authors.

## Materials availability

Authors can confirm that all relevant materials are available on request from the authors.
