# Supplementary figures and images for: NSUN2 promotes osteosarcoma progression by enhancing the stability of FABP5 mRNA via m5C methylation
Source: Cell Death Dis. 2023 Feb 15;14(2):125. doi: 10.1038/s41419-023-05646-x (PMC9932088; doi:10.1038/s41419-023-05646-x)

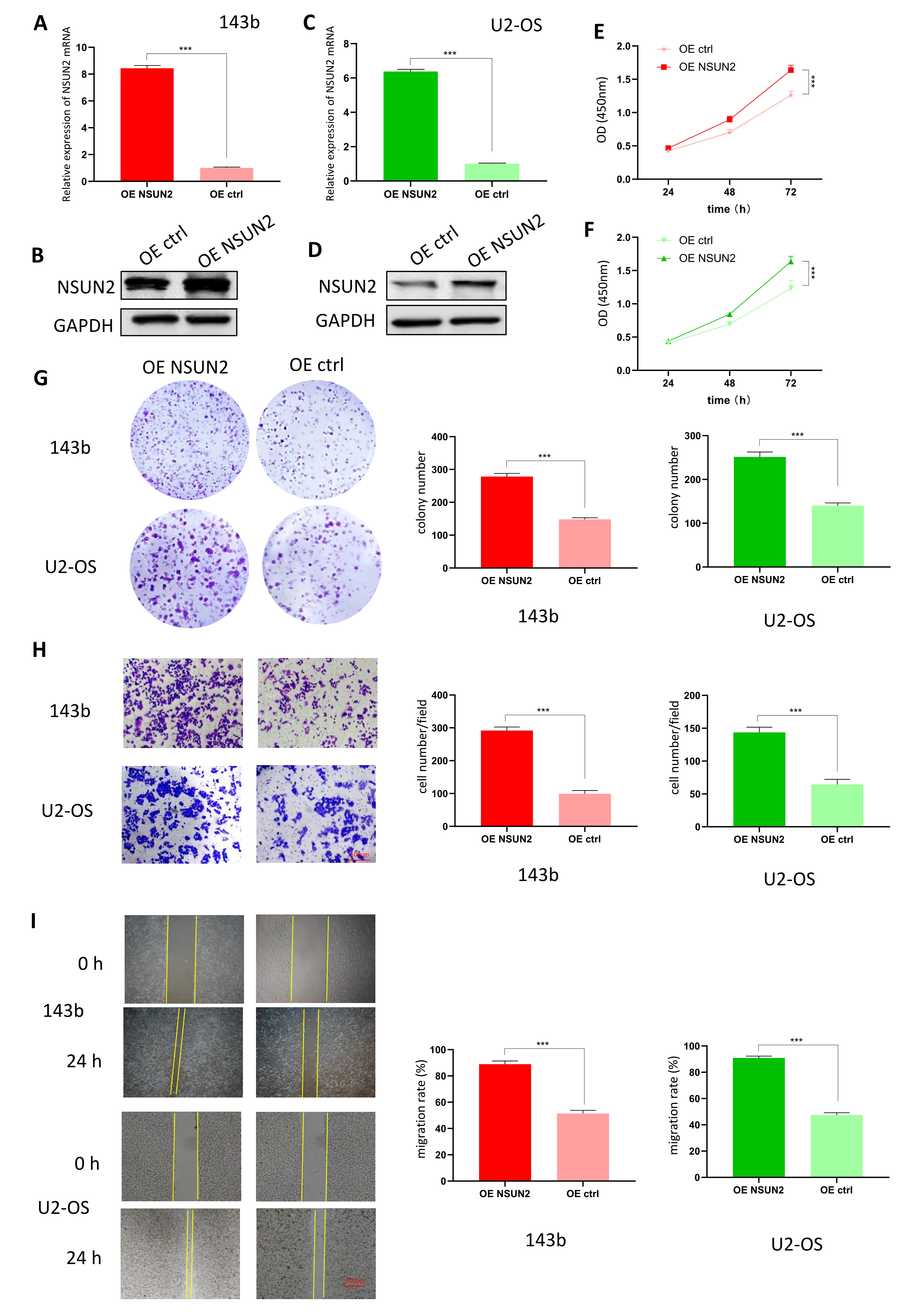

Supplement: Supplementary file 1 — Figure S1 [file 41419_2023_5646_MOESM1_ESM.jpg]

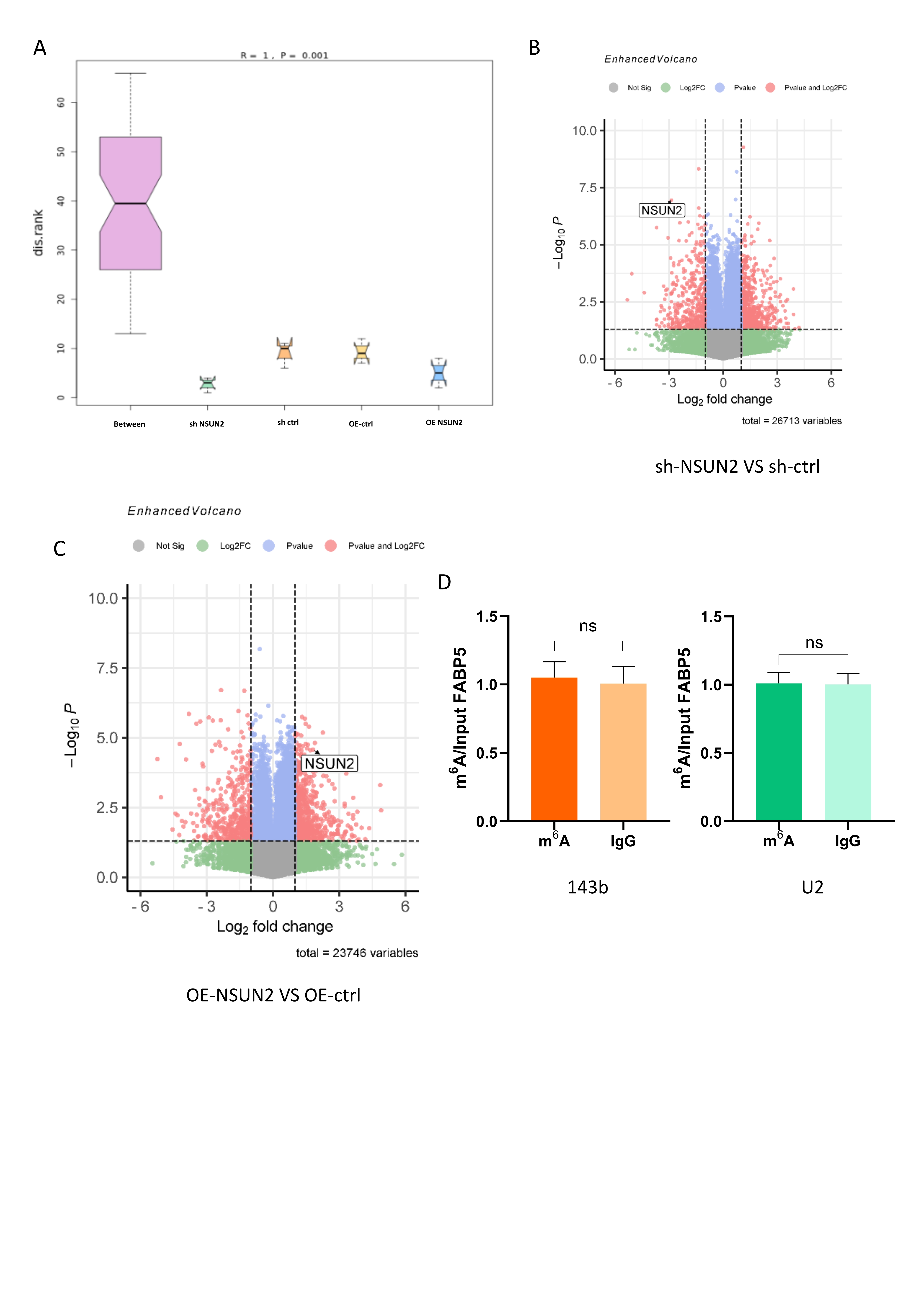

Supplement: Supplementary file 2 — Figure S2 [file 41419_2023_5646_MOESM2_ESM.jpg]

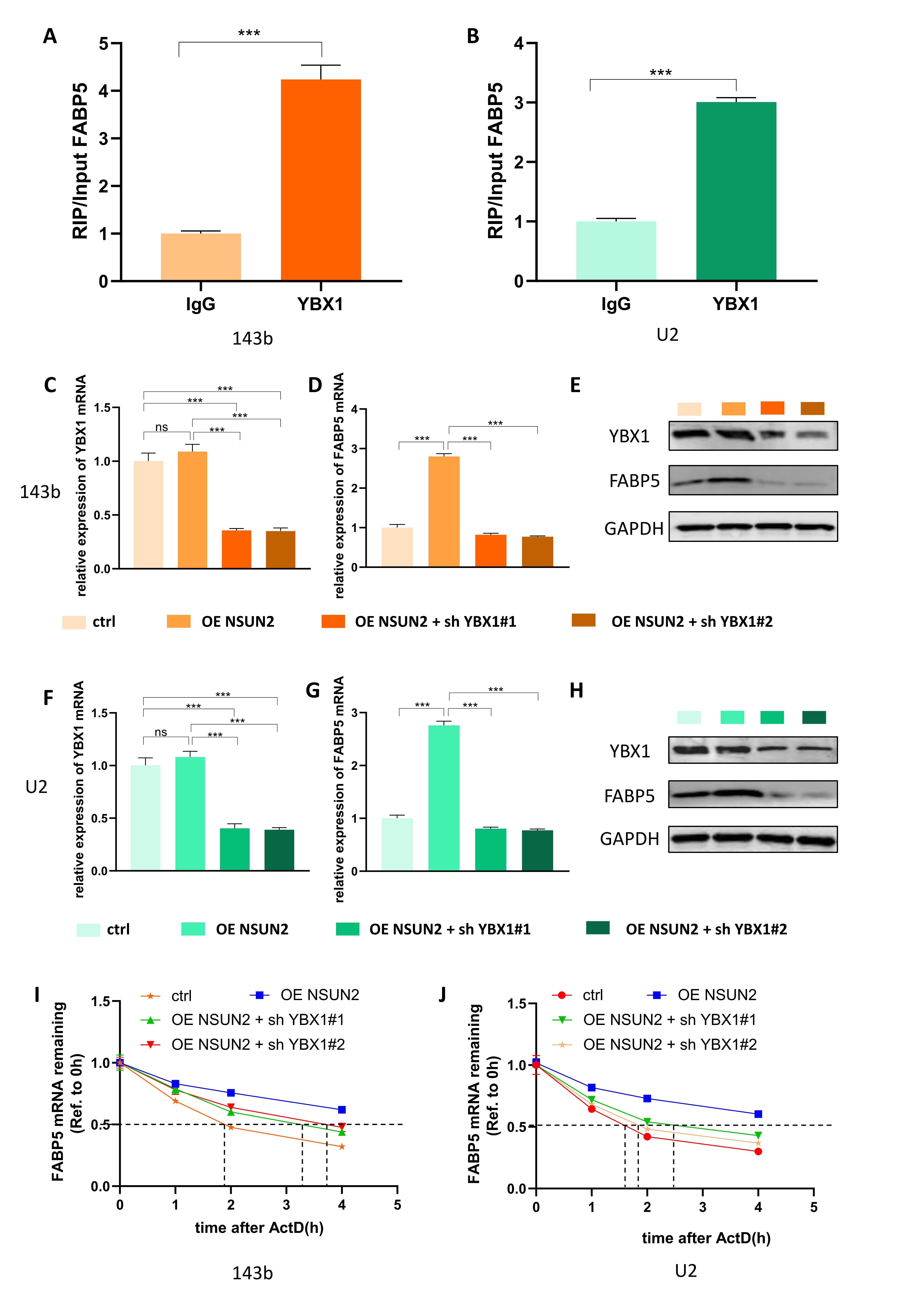

Supplement: Supplementary file 3 — Figure S3 [file 41419_2023_5646_MOESM3_ESM.jpg]

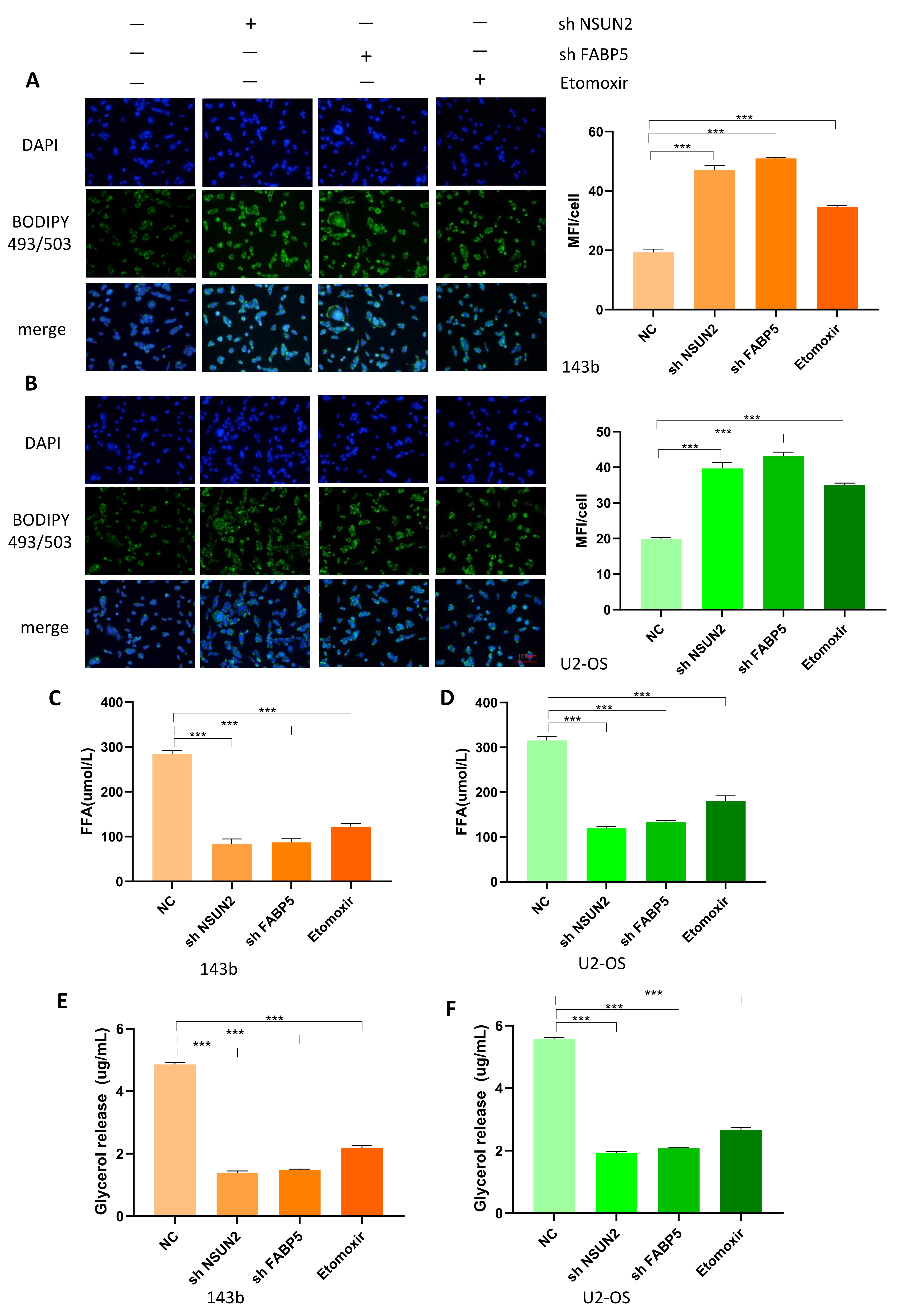

Supplement: Supplementary file 4 — Figure S4 [file 41419_2023_5646_MOESM4_ESM.jpg]

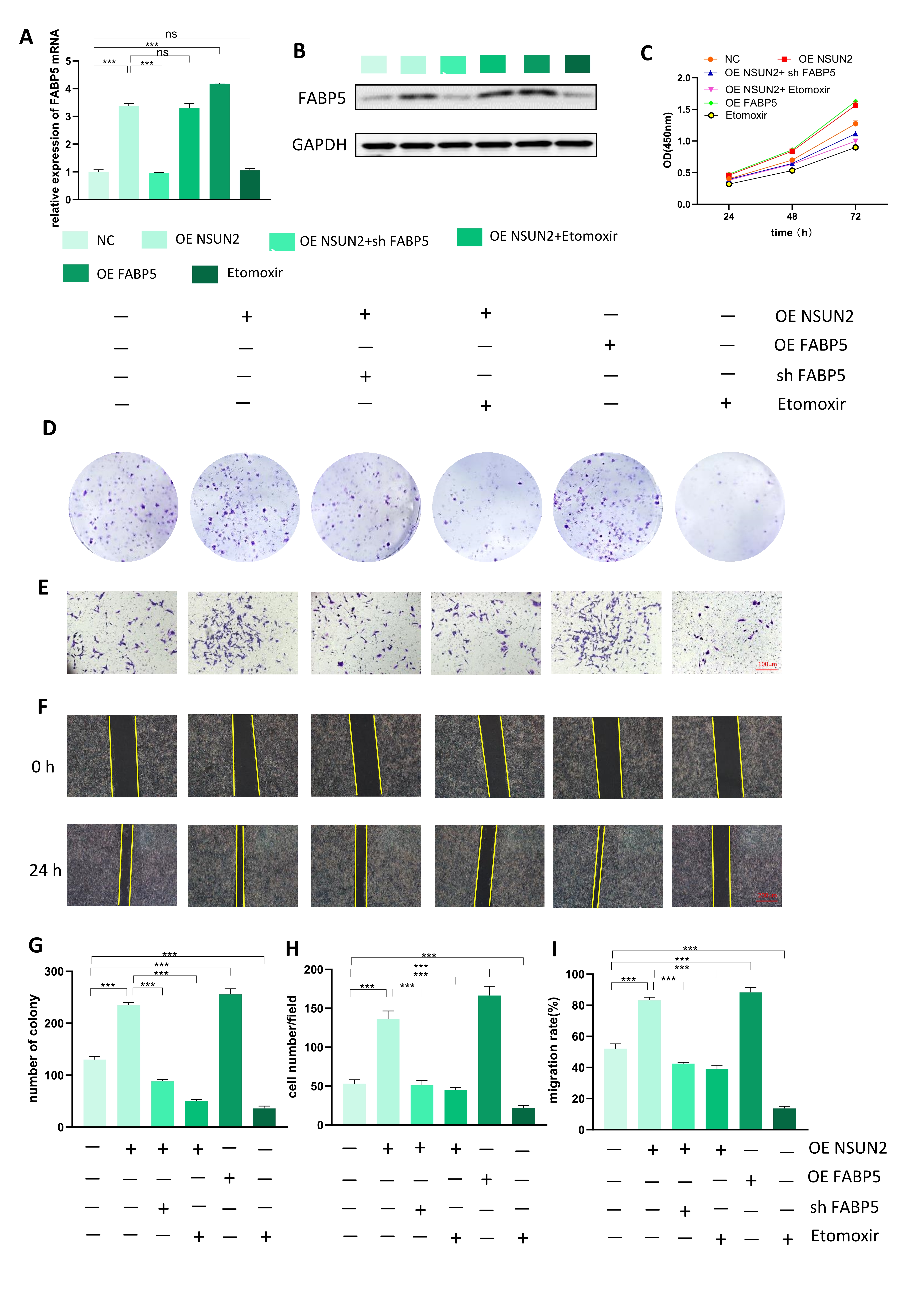

Supplement: Supplementary file 5 — Figure S5 [file 41419_2023_5646_MOESM5_ESM.jpg]

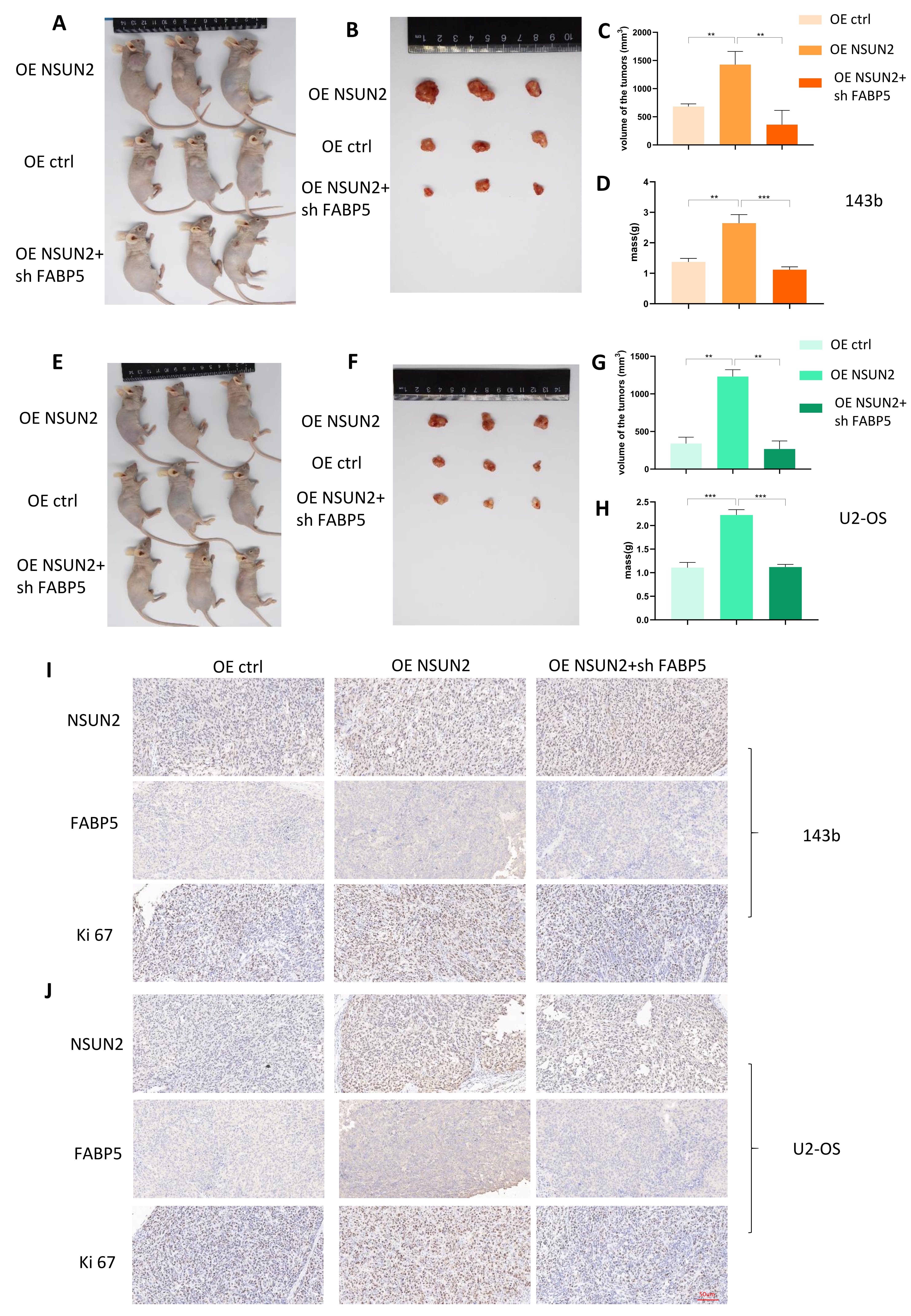

Supplement: Supplementary file 6 — Figure S6 [file 41419_2023_5646_MOESM6_ESM.jpg]
