# Supplementary material for: NSUN2 promotes osteosarcoma progression by enhancing the stability of FABP5 mRNA via m5C methylation
Source: Cell Death Dis. 2023 Feb 15;14(2):125. doi: 10.1038/s41419-023-05646-x (PMC9932088; doi:10.1038/s41419-023-05646-x)
Supplement: Supplementary file 7 — supplementary figure legends [file 41419_2023_5646_MOESM7_ESM.doc]

**Fig. S1** NSUN2 overexpression significantly promotes OS progression in vitro. (A, B) The results of RT-qPCR (A) and western blot (B) showed the expression of NSUN2 in 143b cells. (C, D) The results of RT-qPCR (C) and western blot (D) showed the expression of NSUN2 in U2 cells. (E,F,G) The CCK-8 assay (E, 143b cells; F, U2 cells) and colony formation assay (G) showed that proliferation of OS cells increased when NSUN2 was overexpressed. (H) Transwell assay indicated that the invasion ability of OS cells increased when the expression of NSUN2 increased. (I) The results of the wound-healing assay confirmed that the cells in the OE-NSUN2 group had a higher migration ability. Statistical analysis was performed according to the data of three independent experiments.

**Fig. S2** The data of RNA-seq of stably transfected 143b cells. (A) The result of similarity analysis. (B) The volcano plots of RNA-seq (sh-NSUN2 VS sh-ctrl). (C) The volcano plots of RNA-seq (OE-NSUN2 VS OE-ctrl). (D) The results of m^6^A MeRIP confirmed FABP5 mRNA was not regulated by m^6^A modification in 143b and U2 cells.

**Fig. S3** YBX1 participates in the regulation of NSUN2 on the stability of FABP5 mRNA as the m^5^C reader. (A, B) The results of RIP assay showed YBX1 could bind to FABP5 mRNA in 143b cells (A) and U2 cells (B). (C) The expression of YBX1 mRNA in stably transfected 143b cells. (D) The expression of FABP5 mRNA in stably transfected 143b cells. (E) The expression of YBX1 and FABP5 protein in stably transfected 143b cells. (F) The expression of YBX1 mRNA in stably transfected U2 cells. (G) The expression of FABP5 mRNA in stably transfected U2 cells. (H) The expression of YBX1 and FABP5 protein in stably transfected U2 cells. (I, J) The curve of FABP5 mRNA remaining versus time after ActD treatment in 143b (I) and U2 cells (J).

**Fig. S4** NSUN2 deficiency represses fatty acid metabolism in OS cells. (A, B) The level of neutral lipids and the statistics of the mean fluorescence intensity (MFI) for stably transfected 143b (A) and U2 (B) cells. (C, D) The level of FFAs in stably transfected 143b (C) and U2 (D) cells. (E, F) The level of glycerol in stably transfected 143b (E) and U2 (F) cells. The sh-NSUN2 group means the cells were stably transfected with sh-NSUN2#1. Statistical analysis was performed according to the data of three independent experiments.

**Fig. S5** Both fatty acid oxidation inhibitor and FABP5 deficiency can counterbalance the positive effect of NSUN2 on OS progression. (A, B) The expression level of FABP5 mRNA (A) and protein (B) in U2 cells of each group. (C) The results of CCK-8 assay. (D) The results of colony formation assay. (E) The results of the transwell assay. (F) The results of the wound-healing assay. (G) Quantification and statistics of colony formation assay. (H) Quantification and statistics of transwell assay. (I) Quantification and statistics of wound-healing assay. Statistical analysis was performed according to the data of three independent experiments.

**Fig. S6** FABP5 deficiency can counterbalance the positive effect of NSUN2 overexpression on the proliferation of OS cells in vivo. (A-D) Tumor xenograft models were constructed with stably transfected 143b cells. (E-H) Tumor xenograft models were constructed with stably transfected U2 cells. NSUN2 overexpression could promote the proliferation of OS cells in vivo while sh-FABP5 could offset such promoting effect. (I, J) IHC of NSUN2, FABP5 and Ki-67 in tumors.
