# Supplementary material for: NSUN2 promotes osteosarcoma progression by enhancing the stability of FABP5 mRNA via m5C methylation
Source: Cell Death Dis. 2023 Feb 15;14(2):125. doi: 10.1038/s41419-023-05646-x (PMC9932088; doi:10.1038/s41419-023-05646-x)
Supplement: Supplementary file 8 — the original data of western blot [file 41419_2023_5646_MOESM8_ESM.pptx]

## Slide 1
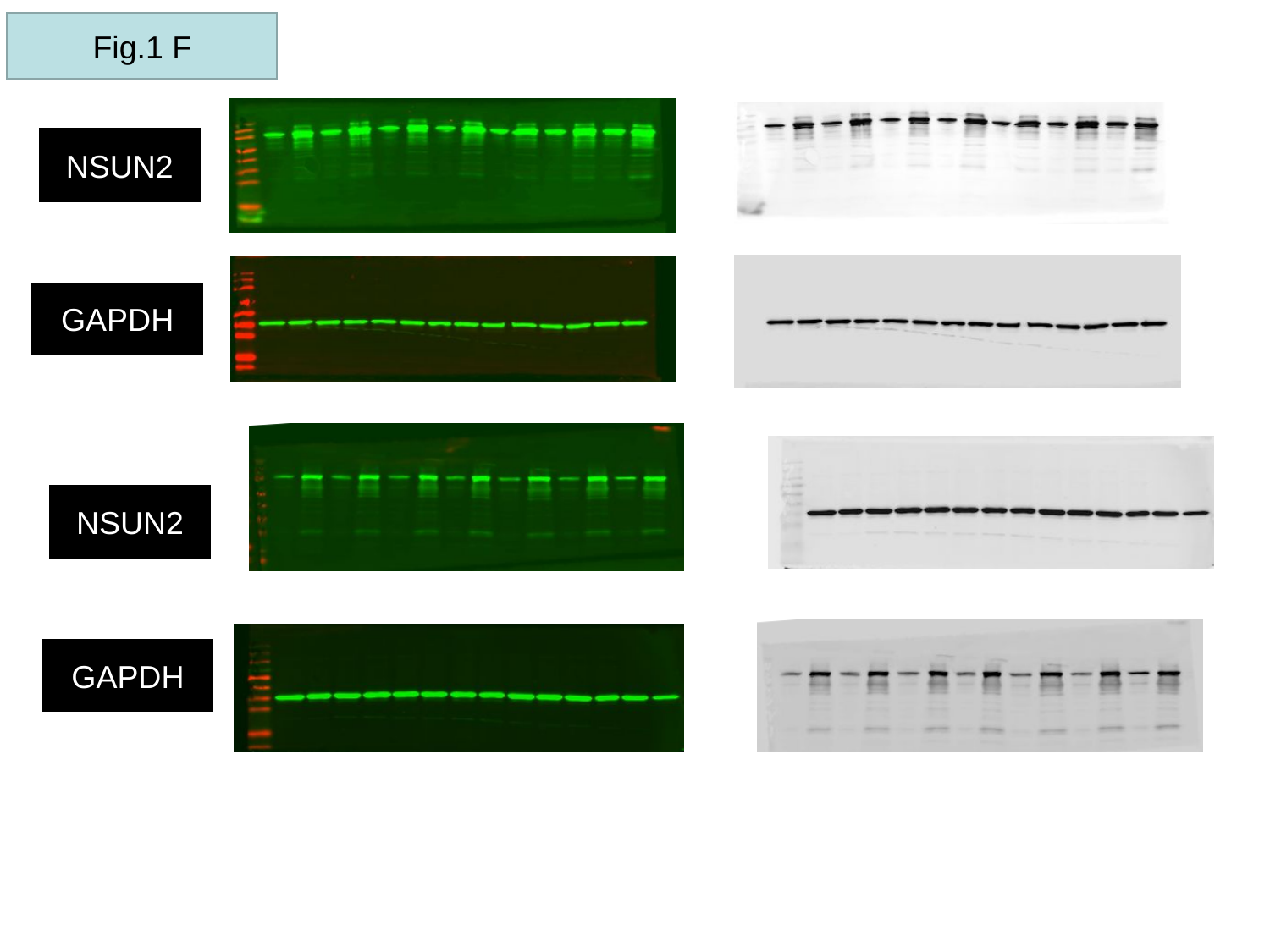

Fig.1 F
NSUN2
GAPDH
NSUN2
GAPDH

## Slide 2
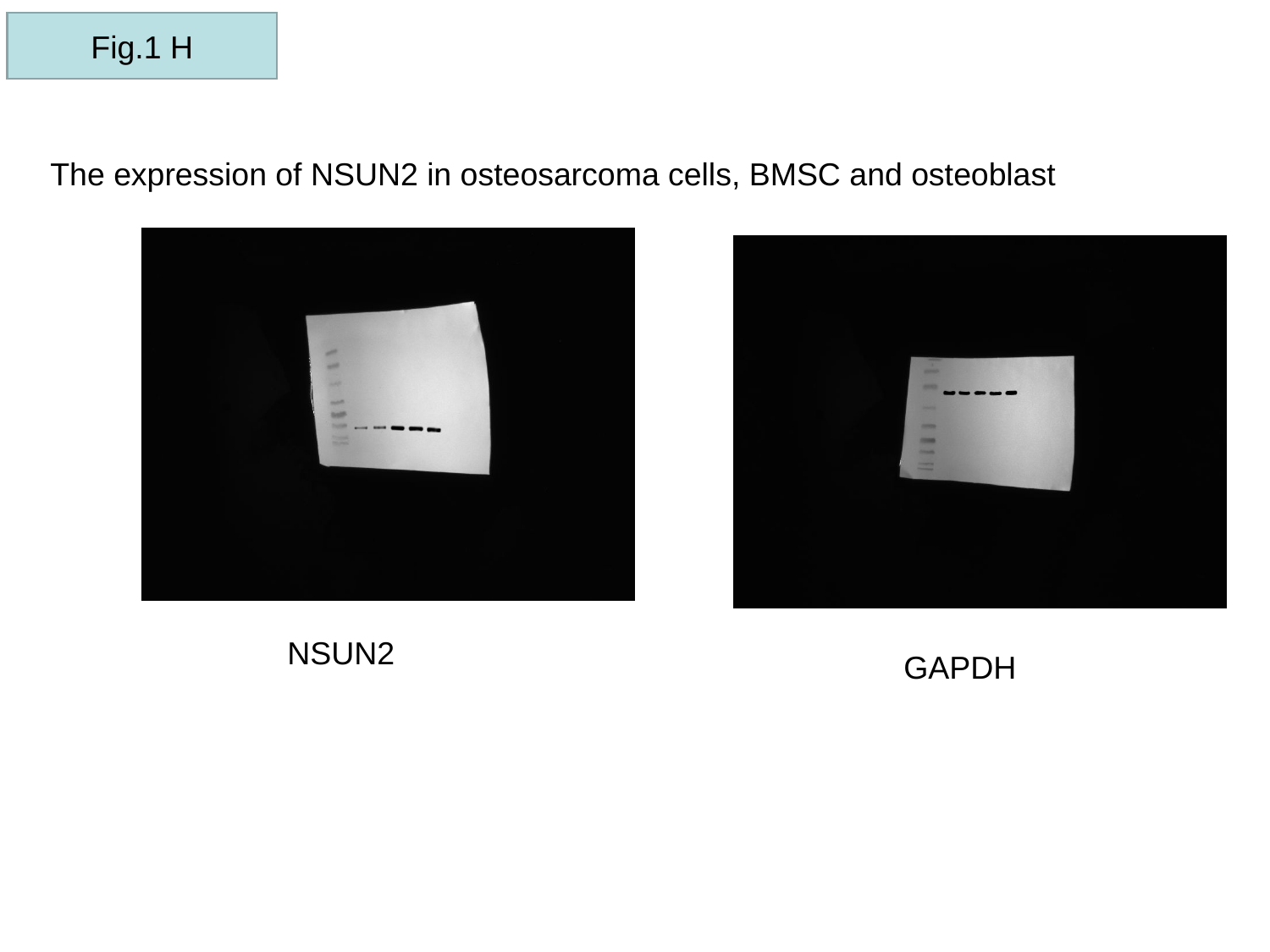

Fig.1 H
The expression of NSUN2 in osteosarcoma cells, BMSC and osteoblast
NSUN2
GAPDH

## Slide 3
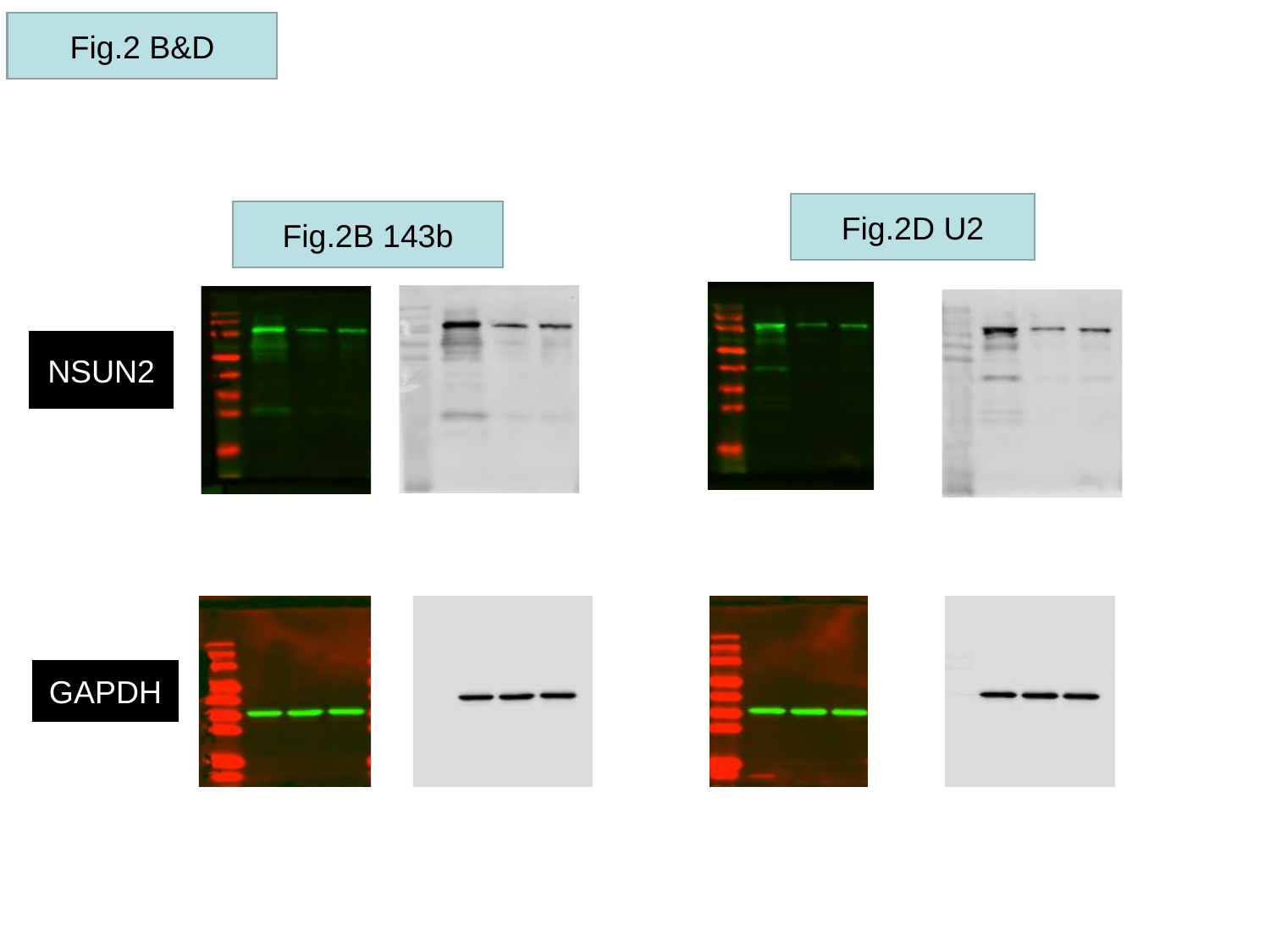

Fig.2 B&D
Fig.2D U2
Fig.2B 143b
NSUN2
GAPDH

## Slide 4
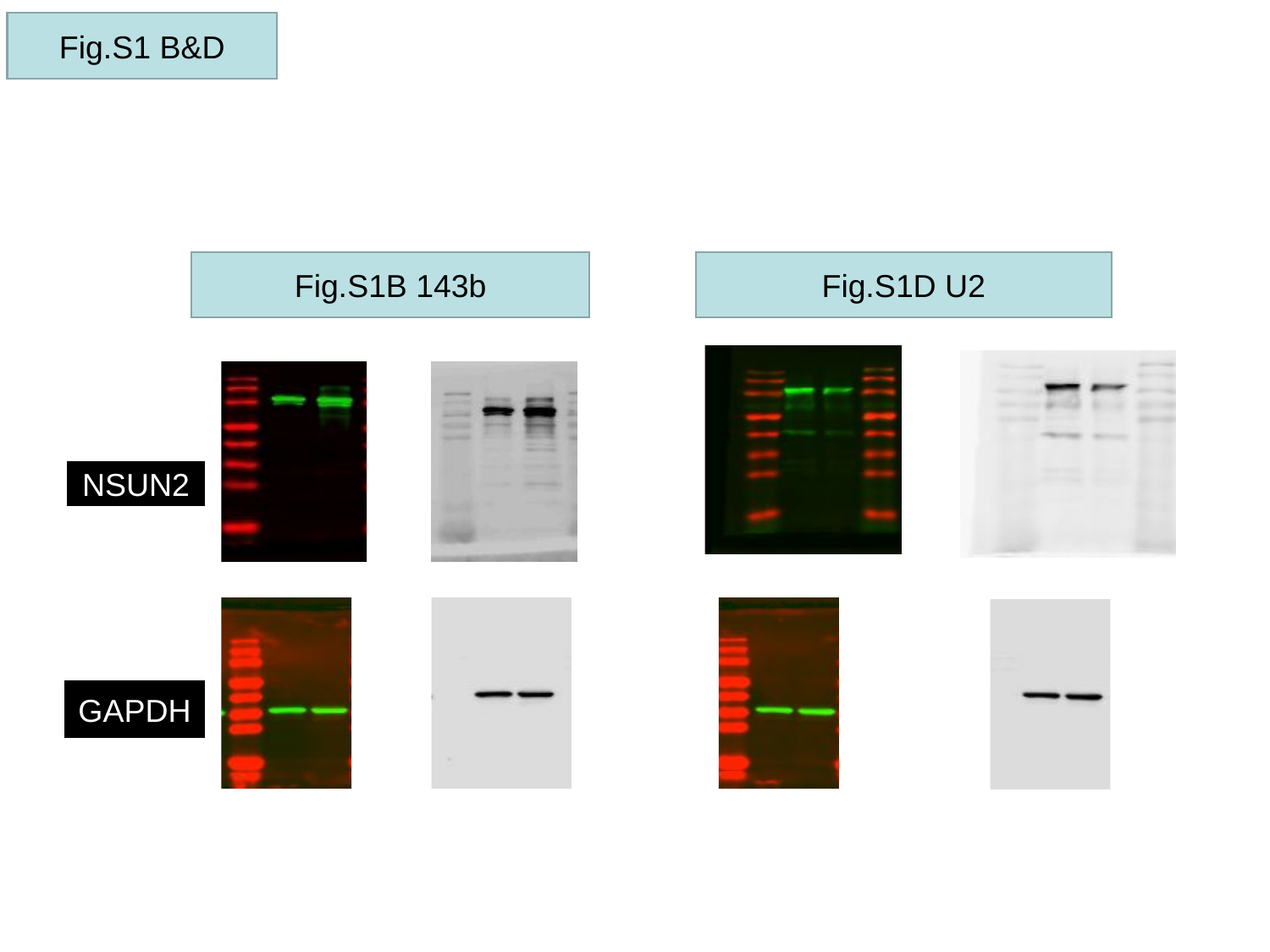

Fig.S1 B&D
Fig.S1B 143b
Fig.S1D U2
NSUN2
GAPDH

## Slide 5
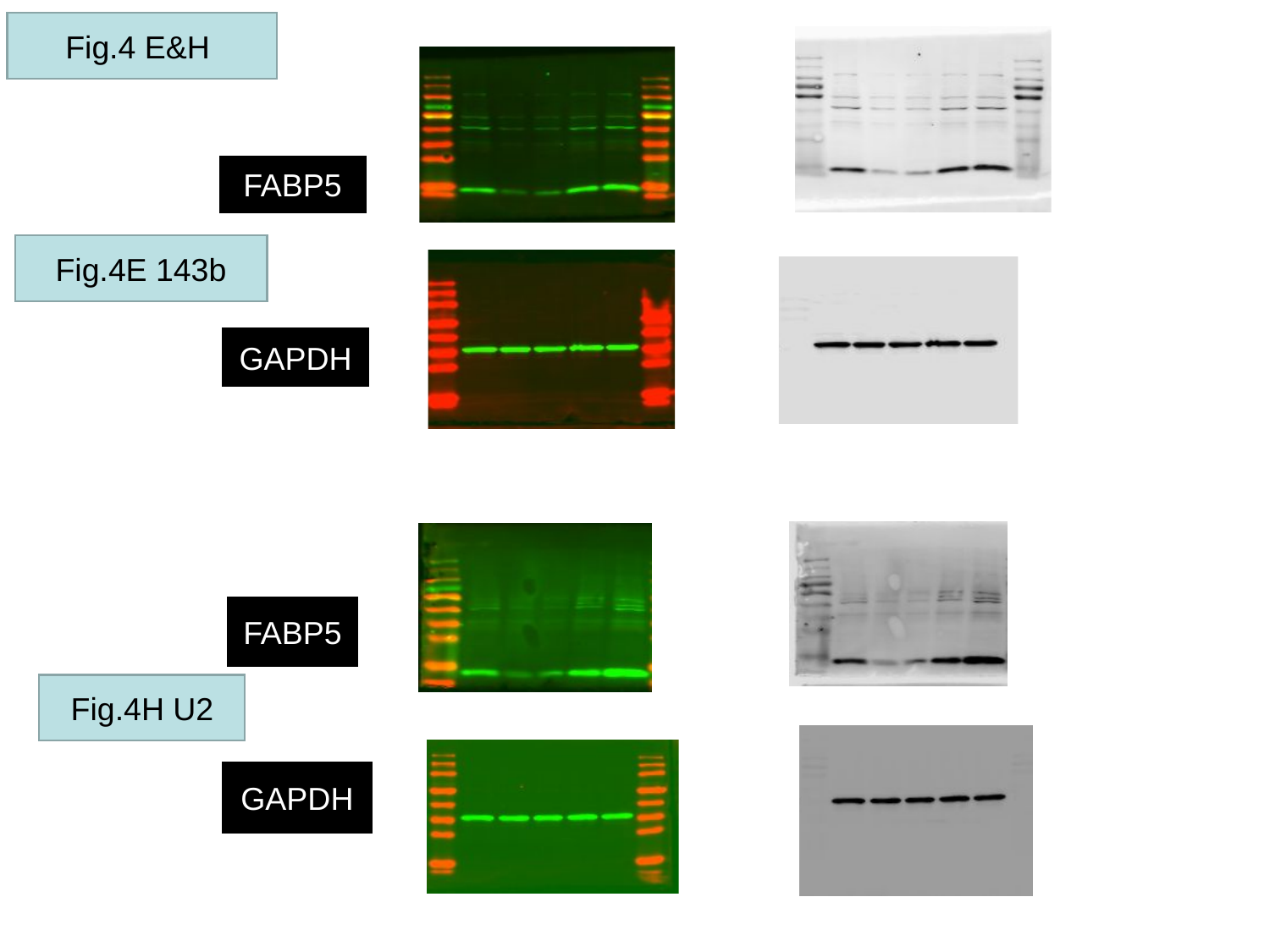

Fig.4 E&H
FABP5
Fig.4E 143b
GAPDH
FABP5
Fig.4H U2
GAPDH

## Slide 6
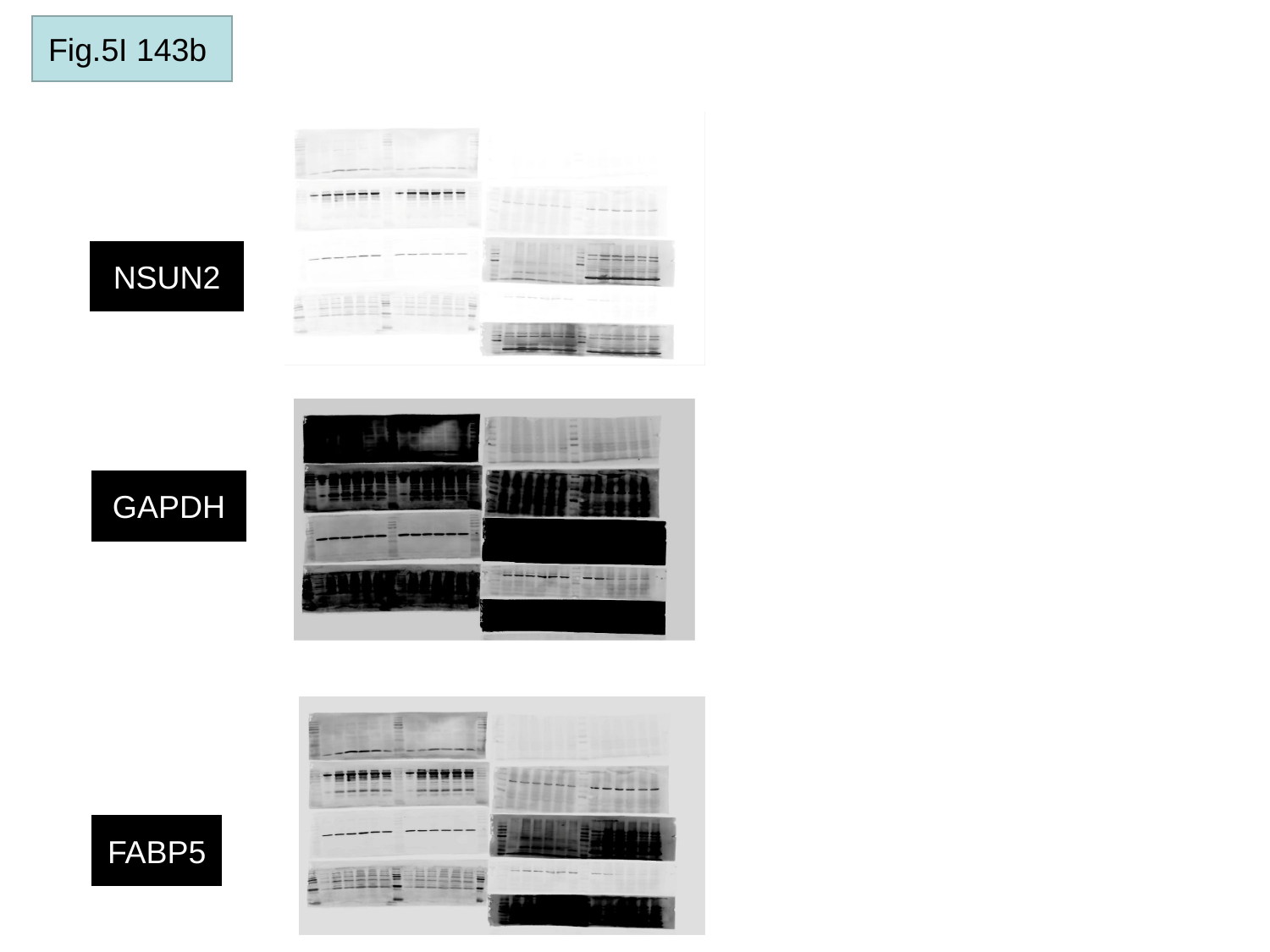

Fig.5I 143b
NSUN2
GAPDH
FABP5

## Slide 7
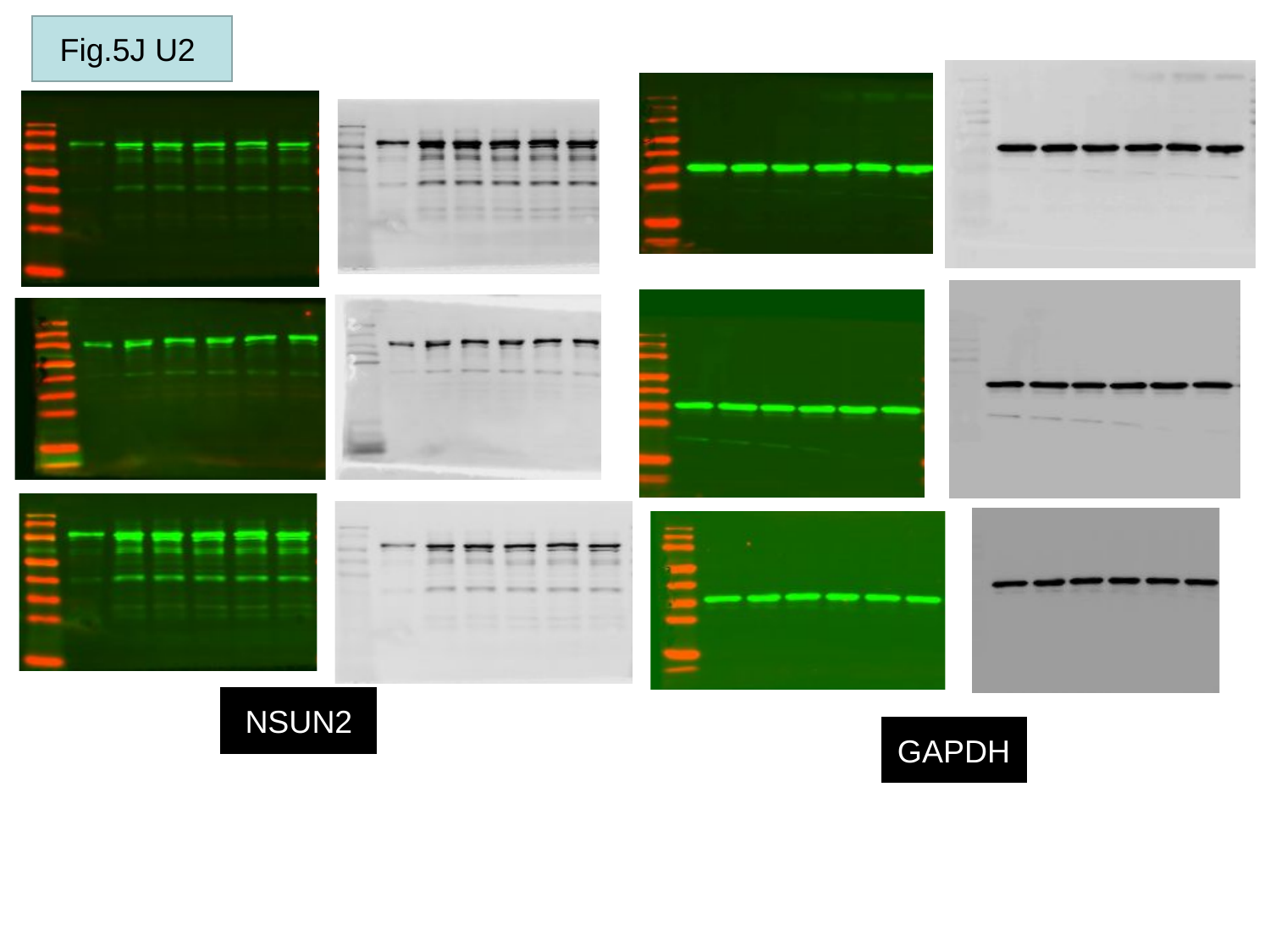

Fig.5J U2
NSUN2
NSUN2
GAPDH

## Slide 8
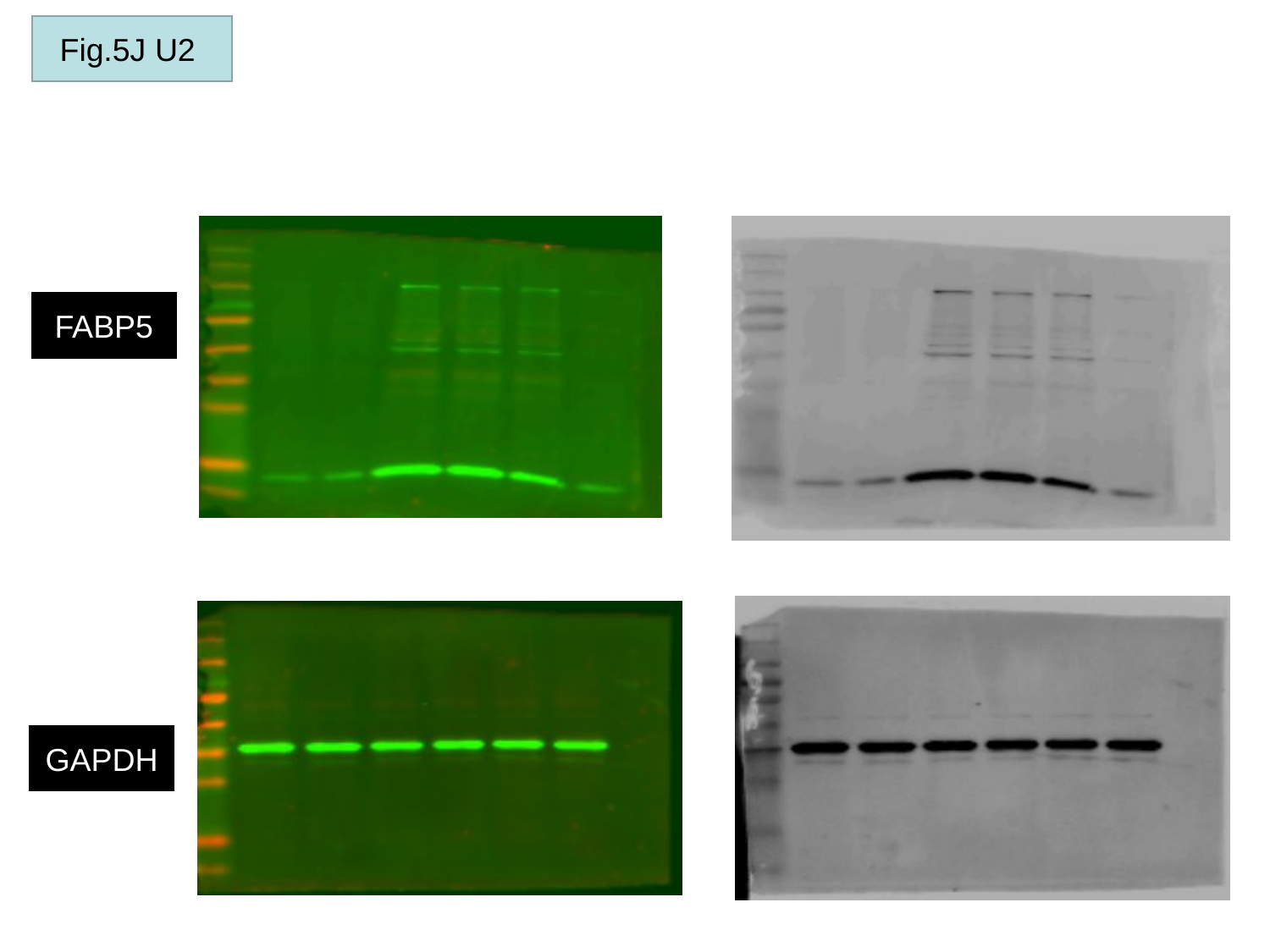

Fig.5J U2
FABP5
GAPDH

## Slide 9
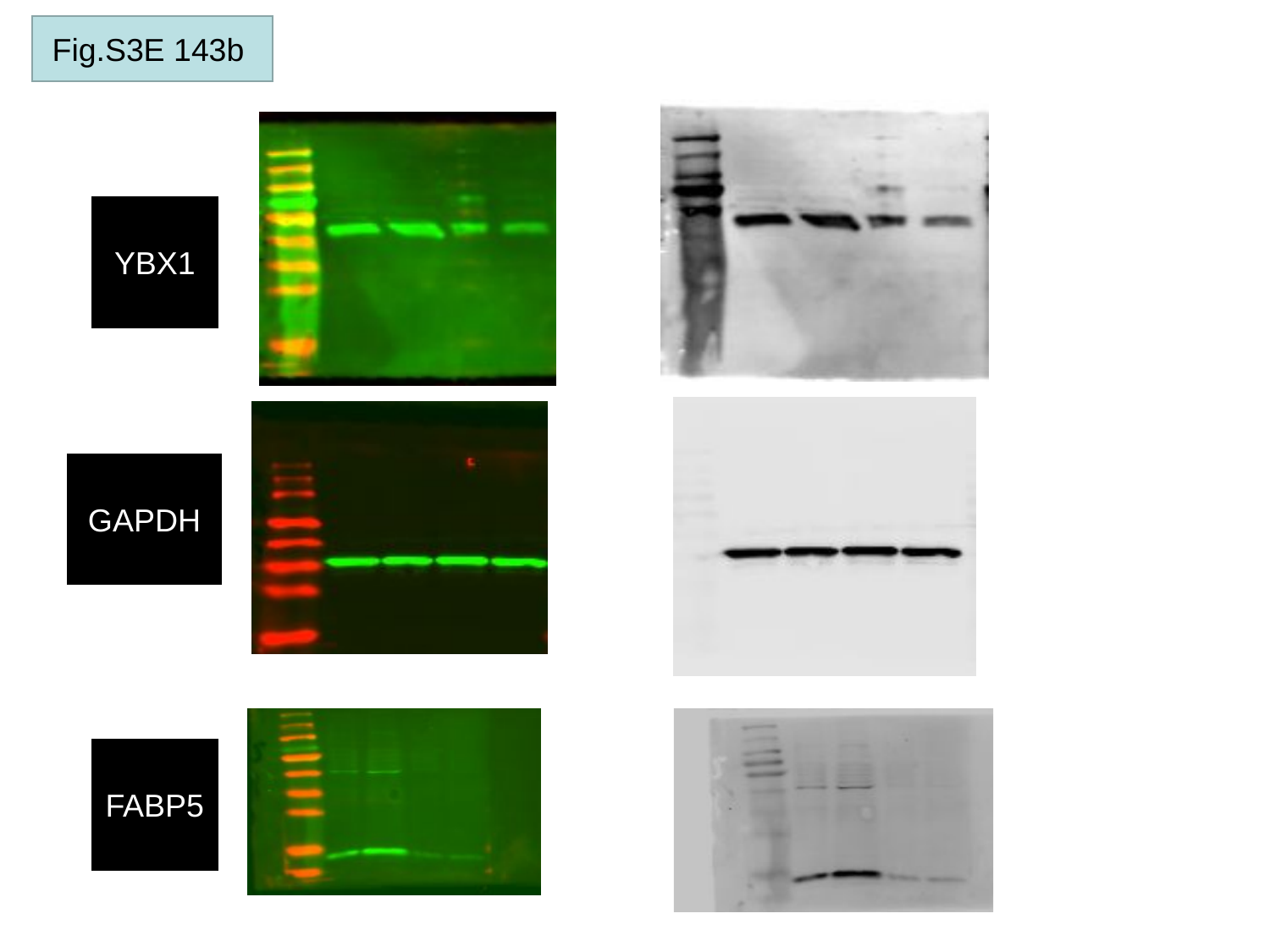

143B
Fig.S3E 143b
YBX1
GAPDH
FABP5

## Slide 10
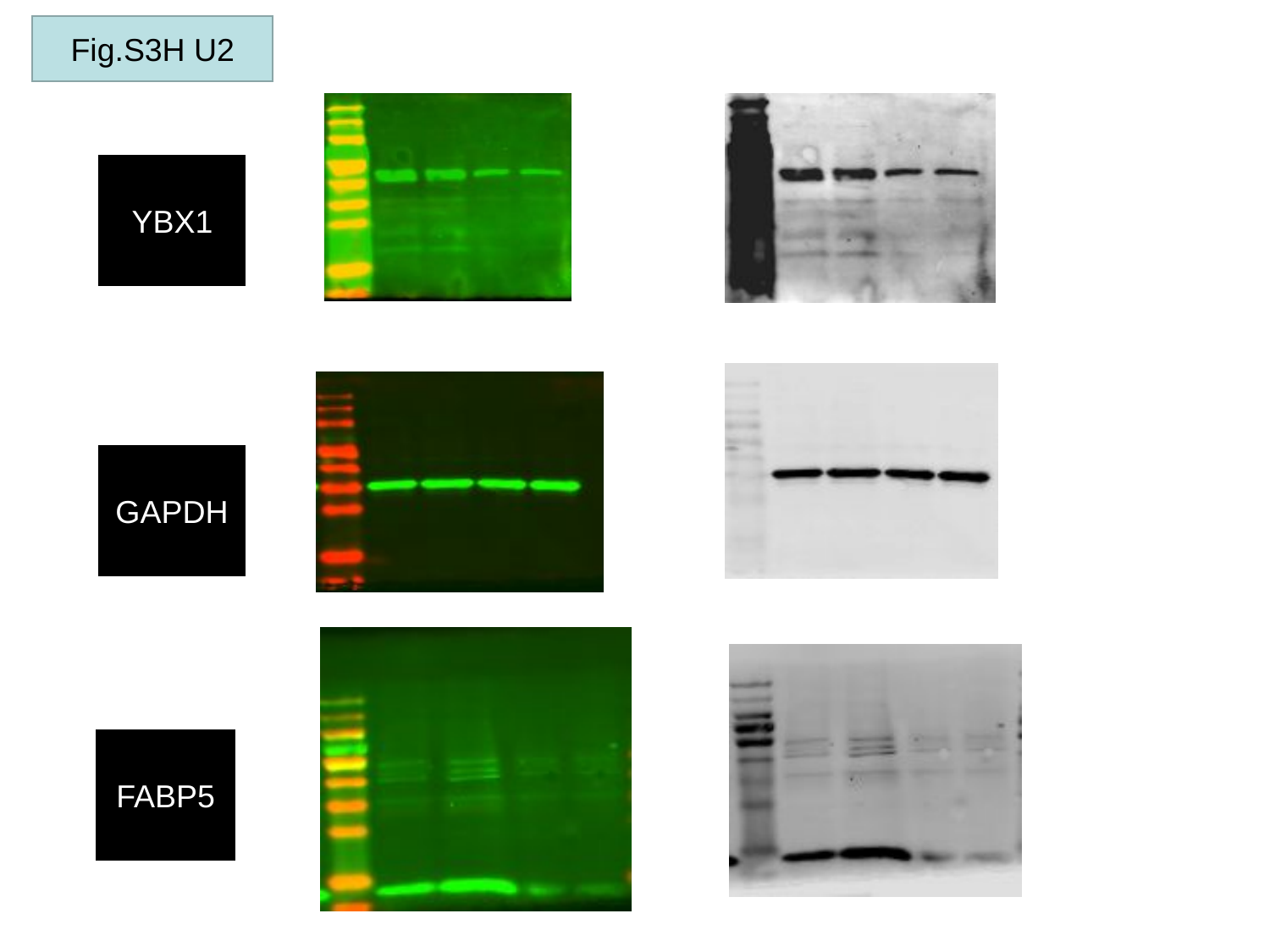

Fig.S3H U2
YBX1
GAPDH
FABP5

## Slide 11
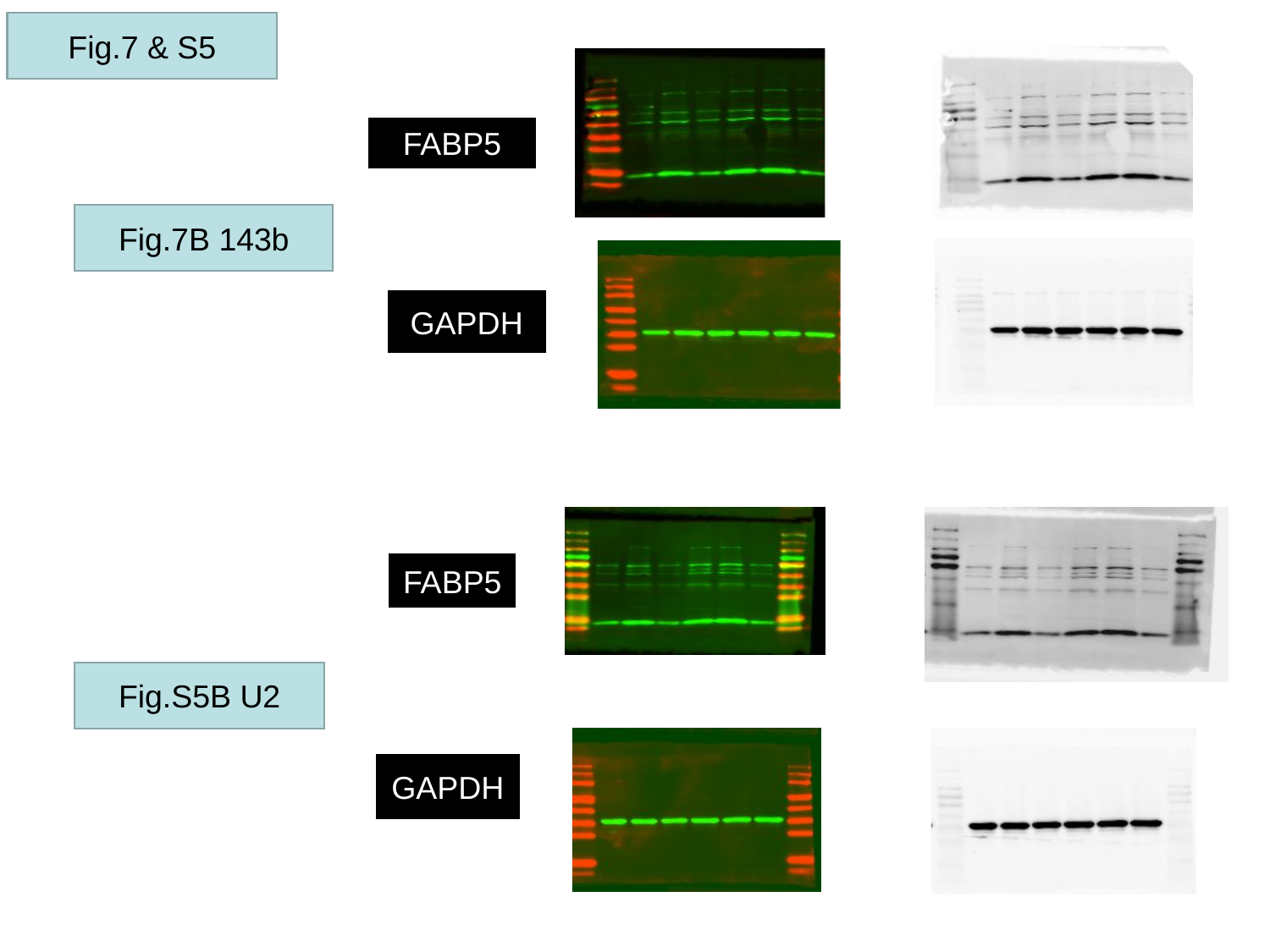

Fig.7 & S5
FABP5
Fig.7B 143b
GAPDH
FABP5
Fig.S5B U2
GAPDH
