# Supplementary material for: NSUN2 promotes osteosarcoma progression by enhancing the stability of FABP5 mRNA via m5C methylation
Source: Cell Death Dis. 2023 Feb 15;14(2):125. doi: 10.1038/s41419-023-05646-x (PMC9932088; doi:10.1038/s41419-023-05646-x)
Supplement: Supplementary file 9 — Author Contribution Statement [file 41419_2023_5646_MOESM9_ESM.doc]

**Author Contributions:** CL, XYL designed and supervised the study; WRX and YM performed the most of cell experiment, animal experiments and wrote the manuscript; LXX and CL were responsible for collecting OS tissues; HS, ZS and ZYF performed a part of the cell experiments; YZQ and ZC collected the information of OS patients.
