# Supplementary material for: NSUN2 promotes osteosarcoma progression by enhancing the stability of FABP5 mRNA via m5C methylation
Source: Cell Death Dis. 2023 Feb 15;14(2):125. doi: 10.1038/s41419-023-05646-x (PMC9932088; doi:10.1038/s41419-023-05646-x)
Supplement: Supplementary file 11 — editing certificate [file 41419_2023_5646_MOESM11_ESM.pdf]

Editing Certificate

This document certifies that the manuscript

NSUN2 promotes osteosarcoma progression by enhancing the stability of FABP5 mRNA via m5C methylation

prepared by the authors

Min Yang, Renxiong Wei, Sheng Zhang, Sang Hu, Xiaoxiao Liang, Zhiqiang Yang, Chong Zhang, Yufeng Zhang, Lin Cai, Yuanlong Xie

was edited for proper English language, grammar, punctuation, spelling, and overall style by one or more of the highly qualified native English speaking editors at AJE.

This certificate was issued on **July 14, 2022** and may be verified on the AJE website using the verification code **E1DE-0E83-2C40-84DD-535P**.

Neither the research content nor the authors' intentions were altered in any way during the editing process. Documents receiving this certification should be English-ready for publication; however, the author has the ability to accept or reject our suggestions and changes. To verify the final AJE edited version, please visit our verification page at aje.com/certificate. If you have any questions or concerns about this edited document, please contact AJE at support@aje.com.

AJE provides a range of editing, translation, and manuscript services for researchers and publishers around the world. For more information about our company, services, and partner discounts, please visit aje.com.
